# Community perspectives on the appropriateness and importance of support goals for young autistic children

**DOI:** 10.1177/13623613231168920

**Published:** 2023-05-18

**Authors:** Hannah Waddington, Hannah Minnell, Lee Patrick, Larah van Der Meer, Ruth Monk, Lisa Woods, Andrew JO Whitehouse

**Affiliations:** 1 Victoria University of Wellington, New Zealand; 2 Autism New Zealand, New Zealand; 3 The University of Auckland, New Zealand; 4 The University of Western Australia, Australia

**Keywords:** autism, early support, goals, neurodiversity

## Abstract

There is little research regarding autistic adult, parent and professional perspectives on support goals for young autistic children. A total of 87 autistic adults, 159 parents of autistic children, and 80 clinical professionals living in New Zealand and Australia completed a survey about the appropriateness and importance of common support goals for young autistic children. Goals related to the adult supporting the child, the reduction and replacement of harmful behaviours, and improving child quality of life were the highest priority across participant groups, while goals related to child autism characteristics, play and academic skills were the lowest priority. Compared to parents and/or professionals, autistic adults gave lower priority ratings for play skills, autism characteristics and participation goals and were more likely to rate these goal domains as inappropriate. More research is needed to understand the underlying reasons for these similarities and differences.

**Lay abstract:**

Researchers do not know much about what autistic adults, parents and professionals think about support goals for young autistic children. People’s views of support goals might also be influenced by their beliefs about early support more generally. This survey involved 87 autistic adults, 159 parents of autistic children and 80 clinical professionals living in New Zealand and Australia. We asked participants questions about themselves and what they thought about early support for young autistic children in general. We then asked participants to rate whether different support goals were appropriate for young autistic children and, if they were appropriate, to rate their level of priority. We found that autistic adults, parents and professionals all rated goals about the adult changing to better support the child, reducing and replacing harmful behaviours and improving the child’s quality of life as the highest priorities. They all rated goals about autism characteristics, play skills and academic skills as the lowest priorities. Compared to parents and/or professionals, autistic adults gave lower priority ratings for play skills, autism characteristics and participation goals. Autistic adults were also more likely to rate goals related to play skills and autism characteristics as inappropriate. While these three participant groups generally agreed on the order of priority of early support goals for young autistic children, autistic adults found goals related to autism characteristics, play and/or participation to be an even lower priority and less appropriate than parents and professionals.

Understandings of autism can vary depending on an individual’s notions of ‘disability’ and their perspectives on early supports (sometimes in the form of interventions, therapies, programmes or services). Autism has historically been viewed through a deficit-focused, medical model lens (Bottema-Beutel et al., 2021; Kapp et al., 2013; Monk et al., 2022; Pellicano & den Houting, 2022). Under this model, autism is perceived as ‘atypical’ or ‘abnormal’ and, thus, a condition which should be changed or ‘intervened’ upon. The medical model is demonstrated in diagnostic criteria, which describe autism as a ‘disorder’ involving difficulties with social communication and the presence of ‘restricted and repetitive’ behaviours, interests and activities (American Psychiatric Association, 2013).

The neurodiversity movement, by contrast, conceptualises autism as a brain *difference*, which involves unique strengths and difficulties and is inherently linked the individual’s identity (Kapp et al., 2013; Pellicano & den Houting, 2022). This movement overlaps with a social model of disability, which proposes that the difficulties experienced by autistic individuals are due to societal stigma and a social environment that is not conducive to the individual’s needs (Kapp et al., 2013; Leadbitter et al., 2021). The biopsychosocial model takes a hybrid approach and postulates that the characteristics of an autistic individual interact with their environment to influence their learning, development and well-being (Engel, 1979). This model could overlap with the neurodiversity movement, depending on the way in which the characteristics of the autistic child are conceptualised. That is, whether the child’s autistic characteristics are conceptualised as ‘atypical’/‘abnormal’ or to be part of the natural spectrum of human development (Bottema-Beutel et al., 2021; Kapp et al., 2013; Pellicano & den Houting, 2022).

Self-advocacy from autistic individuals has greatly contributed to increasing awareness of the neurodiversity movement (den Houting et al., 2021; Leadbitter et al., 2021; Pellicano & den Houting, 2022). Due to this advocacy, many parents are themselves calling for greater access to neurodiversity-affirming supports (Dawson et al., 2022). Professionals are also increasingly aware and responsive to the neurodiversity movement, although training programmes, published research and funding boards often continue to promote medical-leaning models of disability (Dawson et al., 2022; den Houting et al., 2021).

Findings from a large survey indicate that autistic participants were more aware of neurodiversity and more likely to report positive emotions about autism than non-autistic participants (Kapp et al., 2013). Autistic participants were also less likely than non-autistic participants to endorse seeking a ‘cure’ for an autistic child, as were those who were aware of neurodiversity principles. Similarly, findings from another survey in the United Kingdom suggest that professionals may be more likely to refer to autism as a disorder than autistic people (Kenny et al., 2016). In this survey, autistic people and parents were more likely to endorse the use of identity-first language when describing autism (e.g. autistic person), while professionals were more likely to endorse person-first language (e.g. person with autism), indicating that they may perceive autism as separate from the individual (Kenny et al., 2016).

An individual’s support goals for young autistic children may differ according to their model of disability/support. Those who align with a medical model may tend to focus early supports on ‘normalising’ the child and helping them to ‘fit in’ to society by reducing their characteristics of autism (Dawson et al., 2022). Those who align with a social model and/or a neurodiversity framework may be more likely to focus early supports on upskilling those around the child and changing the environment and society as whole in order to increase the child’s quality of life (Gillespie-Lynch et al., 2017; Pellicano & den Houting, 2022). Within these models, goals should also focus on each child’s unique and specific areas of challenge, which could include supporting skills development for everyday tasks, identifying and supporting communication preferences for that individual, and identifying and addressing triggers of aggressive or self-injurious behaviours (Autistic Self Advocacy Network [ASAN], 2021; Dawson et al., 2022; Kapp et al., 2013). Notably, these goals do not relate specifically to autism characteristics and may also apply, for example, to those with other developmental disabilities or delays. Those who align with a biopsychosocial model may endorse goals related to both teaching the child skills and changing the environment around the child (Engel, 1979).

Several studies have examined parents’ specific support goals for their autistic children (Ghanadzade et al., 2018; Pituch et al., 2011; Rodger et al., 2004). Rodger et al.’s (2004) study involved 22 parents of young autistic children who were participating in an in-home support programme in Australia. Parents collaborated with facilitators to set priority goals for their children. Most high-priority goals related to communication, followed by ‘decreasing socially unacceptable behaviour, such as tantrums, screaming or replacing any such behaviour with a more socially acceptable behaviour’, and then social interaction (p. 36). Pituch et al.’s (2011) online survey examined the support priorities of 90 parents of autistic children living in New Zealand, Australia, the United States or Canada. The highest rated priorities pertained to aspects of social interaction (e.g. makes friends), communication (e.g. describes events/feelings) and safety (e.g. pedestrian safety skills). Finally, Ghanadzade et al. (2018) conducted a survey of 207 Iranian parents whose children were attending ‘rehabilitation centres’ in Tehran. Five of their 10 highest priorities related to communication, while the remaining goals pertained to safety, academic skills, self-care and social skills. As a whole, regardless of when or where the study was conducted, these results suggest that parents tend to prioritise goals targeting communication, social interaction and safety. Due to an absence of scholarly research, it is not possible to formally confirm whether these parent goals align with, or differ from, the priorities of autistic adults and professionals.

Autistic adults, parents and clinical professionals each have distinct perspectives on support goals for young autistic children. Autistic adults have unique insight into the experiences of autistic children, while parents and clinical professionals are often involved in setting and prioritising support goals. However, there does not appear to be any research examining perceptions of support goals for young autistic children across these three groups. This study involved a survey of autistic adults, parents and clinical professionals living in New Zealand and Australia regarding their perceptions of various support goals for young autistic children. The study examined the reported priority-level and appropriateness of each type of support goal across participant groups. It also examined whether priority-level for each type of goal was associated with participant demographic characteristics and their perspectives on early supports for autistic children, including their model of disability/support (e.g. a medical model or a social model). Finally, the study examined participants’ perceptions of early support and the likelihood that a participant rated goals as inappropriate differed between autistic adults, parents and professionals.

Based on previous research, it was hypothesised that autistic adults would be more likely than parents and professionals to align with models of disability/support that are based on the principles of neurodiversity, such as the social model (Gillespie-Lynch et al., 2017; Kapp et al., 2013; Pellicano & den Houting, 2022). It was also hypothesised that they would be more likely to endorse and prioritise goals related to modifying the environment around the child (including through upskilling the adults who support them) and improving quality of life. Similarly, it was hypothesised that autistic adults would be less likely than parents and professionals to endorse and prioritise goals that could be conceived as trying to ‘normalise’ the child and particularly those goals aimed at reducing characteristics of autism.

## Methods

### Participants

Individuals were eligible to participate in the survey if they were living in New Zealand or Australia and were: (a) an autistic adult over the age of 18 years, (b) a parent/caregiver (referred to herein as parent) of an autistic child under 18 years or (c) a professional working with autistic children under 6 years. While not an explicit exclusion criterion, individuals who did not have the ability to read and complete the survey without assistance would likely not have been able to participate.

### Materials

The survey was co-designed by all authors except R.M., who became involved after the survey closed. It is included as Supplementary Document 1. The early support goals included in the survey were informed by the authors’ own knowledge of early development, the skills included in common developmental assessments (e.g. Mullen, 1995; Rogers & Dawson, 2010; Sparrow et al., 2016) and New Zealand-specific holistic frameworks of learning and development (Carr & May, 2008; Rochford, 2004). The latter were included in an attempt to reflect Indigenous goal priorities alongside Western priorities.

All authors, except R.M., initially met twice over Zoom to mutually decide on the aims of the survey and relevant resources to inform its development. The draft of the survey was compiled by H.M. and sent to all remaining authors as well as other members of the autistic and autism communities (see section ‘Community involvement statement’) for feedback. H.M. then attempted to address all feedback, either by including the edit in the survey or explaining why the suggested edit was not possible (e.g. it was beyond the scope of the survey). All authors then reviewed the revised survey and either approved the version or suggested further revisions. All authors were satisfied with the final version of the survey.

#### Survey content

The survey had three sections: (1) demographic information, (2) perspectives on early supports and (3) appropriateness and priority-level of early support goals. Participants could select ‘prefer not to say’ for any question they did not wish to answer. Sections (2) and (3) contained several free-text boxes for participants to expand on their responses. These responses will be analysed separately to this study.

Participants were required to provide consent to participate before they could begin answering the questions. The demographic section (section 1 in Supplementary Document 1) began with screening questions related to participants’ location and identity/role. The following demographic information was collected for all eligible participants: (a) gender, (b) ethnicity, (c) the highest level of formal education, (d) experience with young children and (e) identity/role (autistic adult, parent and/or professional). Although participants may have had more than one identity/role (e.g. an autistic professional), they were instructed to select one primary identity/role for the completion of the survey.

Further demographic information gathered from autistic adults included their age, whether they were formally diagnosed, age of autism diagnosis (if applicable), additional diagnoses and what autism-specific support they had received (if any), when they were 5 years and younger. Parents were asked to detail their relationship to the autistic child and their child’s age, age of diagnosis, gender, ethnicity and additional diagnoses, as well as whether their child received any supports currently and/or when they were 5 years old or younger. Professionals were asked about their role, how long they had been in that role and their years of experience working with autistic children.

In section 2 in Supplementary Document 1, all participants were asked if it was appropriate to provide early support to preschool-aged autistic children. Those who believed it was appropriate were provided with a statement about the purpose of early supports and selected all statements that aligned with their beliefs (see Supplementary Document 1 for a list of statements). Finally, all participants were asked to select the statement that best aligned with their model of disability/support. These statements are detailed in Supplementary Document 1 and were based on medical, social and biopsychosocial models and an approach based on the child’s unique strengths and difficulties, informed by the neurodiversity framework (Kapp et al., 2013; Leadbitter et al., 2021).

In section 3 in Supplementary Document 1, participants rated the appropriateness and priority-level of different early support goals. These goals were grouped into 10 overarching goal domains, which each consisted of two to seven specific goals. These domains were: (a) autism characteristics, (b) communication, (c) academic skills, (d) motor skills, (e) play skills, (f) daily living skills, (g) participation, (h) reducing harmful behaviours, (i) quality of life and (j) goals for the adults who support the child, including parents, other family members, teachers, other professionals or members of the community (see Supplementary Table S3 for a list of goals). Participants were asked to ‘imagine that each goal is something that a 3-year-old autistic child is developmentally able to do but is not doing most of the time’. Participants indicated whether each goal was appropriate (dichotomous) and then rated ‘appropriate’ goals on a 4-point Likert-type scale from high priority (1) to not at all a priority (4). Participants could also select ‘no opinion/not sure’. Cronbach’s alpha for adult supports was 0.66 and exceeded 0.8 (range = 0.81–0.96) for all remaining goal domains.

### Procedures

#### Timeline

The survey went through several rounds of feedback (see section ‘Community involvement statement’) and was hosted on Qualtrics between 16 January 2022 and 11 February 2022. Anonymous survey responses were then downloaded from the Qualtrics database to a password-protected laptop.

#### Distribution methods

The survey was distributed online through autism support organisations and social media pages for autism support groups for autistic adults, parents and/or professionals in New Zealand and Australia. Snowball sampling was used, as participants were invited to share the survey link with others.

### Data management

Responses were only analysed for those participants who completed at least one question within the *early support* section. Some categories were combined for the inferential analyses to reduce the number of small groups. Ethnicity was collapsed into five categories: New Zealand/Australian European, Māori, Asian, Pacific Peoples and ‘Other’. To prioritise Māori within the sample, ethnicity was coded as Māori if this was one of participants’ stated ethnicities (Cormack & Robson, 2010). Otherwise, all participants who had ethnicities from two or more of these categories were coded under ‘other’. The highest level of formal education was collapsed into five categories: some or all of high school, trade, diploma/undergraduate-level study, postgraduate-level study and other, which encompassed responses that did not fit in any category, ‘prefer not to say’ and ‘primary education only’. Participants’ relationship with the child was also reduced to three categories: mother, father and other family member/guardian.

The Likert-type responses for each support goal were first coded dichotomously in terms of whether or not the participant considered the goal to be inappropriate. Next, a priority score was given, with ratings of both ‘not an appropriate goal’ and ‘an appropriate goal but not a priority’ coded the lowest priority. Finally, the mean priority scores were calculated across all items within each goal domain. In line with previous research, mean scores were not calculated in instances where participants had not completed or had responded ‘no opinion/not sure’ to at least one item within a particular domain (Jonkman et al., 2023). Thus, this score was missing for these participants in all relevant analyses. The analyses in Supplementary Table S2 indicate that the participants who had missing data in at least one domain were more likely to prefer not to disclose their level of education compared to those who did not have any missing data. Aside from this, there were no significant differences.

### Data analysis

Descriptive statistics were calculated for all universal and identity/role-specific (unique) demographic characteristics, participants’ perspectives on early supports and the appropriateness and priority-level of each support goal and goal domain. Analyses were performed using IBM SPSS statistics. All test-statistics and *p*-values are reported in tables, rather than in-text. The *adult supports, reducing harmful behaviours* and *quality of life* were excluded from all relevant analyses because ⩾90% of participants gave priority scores of <2 in each of these domains so there was limited variation. Statistical analyses were conducted separately for seven remaining goal domains for each relevant research question. As such, the Bonferroni correction has been applied to all analyses related to support goals to control for type 1 error, with *p* ⩽ 0.007 (0.05/7 domains) being statistically significant. Each specific approach to statistical analysis is described under its corresponding research question.

#### Is there an association between participant group and perceptions of early support?

It was hypothesised that participant groups (parents, professionals, autistic adults) would differ in their perceptions of early supports. This was examined using two separate Chi-square analyses with post hoc Bonferroni-adjusted pairwise comparisons (adjusted *p* ⩽ 0.05). The first analysis pertained to the proportion of participants who believed that early support was appropriate according to participant group and the second pertained to the proportion of participants who aligned with each model of support according to participant group. The ‘not appropriate’ category was excluded from analysis for *appropriateness of supports* because too few participants gave this response. For all remaining analyses, the expected value was 5 or greater in at least 80% of cells.

#### Is there an association between overall demographic characteristics, perspectives on early supports and support goal priorities?

It was hypothesised that certain participant demographic characteristics, including participant identity/role, and perspectives on early support would predict support goal priorities. This was examined using hierarchical linear regressions. Predictors were entered in two blocks. The first block contained all universal demographic characteristics including participant primary identity/role, country of residence, gender, participant ethnicity, level of formal education and experience with young children. The second block contained participants’ perspectives on early supports including their model of support and whether they thought the provision of early support was appropriate. The residuals for all analyses were approximately normally distributed.

#### Is there an association between unique demographic characteristics and support goal priorities?

It was hypothesised that certain demographic characteristics that were unique to each participant group (e.g. parent relationship to child and professional role) would predict support goal priorities. This was examined using separate multiple regressions for each participant group and support goal domain. Furthermore, to ensure that the regression was adequately powered, only variables with ⩾10 participants were included (VanVoorhis & Morgan, 2007), so some professional roles were not analysed, nor were some co-occurring conditions, or types of support reported by autistic adults or parents in relation to their autistic child. Residuals for *communication* for parents, *autism characteristics* and *participation* for autistic adults and *communication* and *participation* for professionals were approximately normally distributed following the Box–Cox transformation. The residuals for all remaining analyses were approximately normally distributed.

#### Is there an association between participant group and the appropriateness of support goals?

It was hypothesised that participant groups would differ in their perceptions of the appropriateness supports goals. This was examined using the Chi-square analyses with post hoc pairwise comparisons (*p* ⩽ 0.007) to determine whether the proportion of participants who found at least one goal in each goal domain to be inappropriate differed across participant groups.

### Community involvement statement

This research was co-designed at all stages by H.W., H.M., L.P., L.v.D.M. and A.W. This included the development and dissemination of the survey, interpretation of the results and the write-up of the final study. L.P. and R.M. are autistic adults. L.P. and L.v.D.M. are employed by a national autism organisation. H.W. is related to autistic young people. A larger advisory group of autistic adults, parents and professionals provided input on the contents of the survey, as did a post-doctoral fellow, and a mother of an autistic child.

## Results

### Demographic characteristics

A total of 368 individuals consented to participate in the survey. Of these, two did not complete the eligibility questions, 22 did not meet eligibility criteria and 18 did not complete the demographic characteristics and/or the perspective on early support sections. This resulted in useable data for 326 participants including in terms of primary identity/role, 87 autistic adults, 159 parents of autistic children and 80 professionals. Demographic characteristics common to all participants are summarised in Table 1, while characteristics for each unique participant group are summarised in Supplementary Tables S4–S6.

**Table 1.** Demographic characteristics for autistic adults (*n* = 87), parents (*n* = 159) and professionals (*n* = 80).

Demographic characteristic	All *n* (%)	Autistic adults *n* (%)	Parents *n* (%)	Professionals *n* (%)
All identities/roles*				
Autistic adult only	66 (20%)			
Parent only	132 (40%)			
Professional only	78 (24%)			
Autistic parent	32 (10%)			
Autistic adult and professional	3 (1%)			
Parent and professional	10 (3%)			
Autistic parent and professional	5 (2%)			
Country				
New Zealand	239 (73%)	52 (60%)	133 (84%)	54 (67%)
Australia	87 (27%)	35 (40%)	26 (16%)	26 (33%)
Gender				
Male	39 (12%)	16 (18%)	14 (9%)	9 (11%)
Female	270 (83%)	56 (64%)	144 (90%)	70 (88%)
Gender diverse	17 (5%)	15 (17%)	1 (1%)	1 (1%)
Ethnicity				
NZ/Australian European	230 (71%)	64 (74%)	109 (69%)	57 (71%)
Māori**	35 (11%)	10 (11%)	21 (13%)	4 (5%)
Asian	20 (6%)	4 (5%)	10 (6%)	6 (8%)
Pacific Islander	5 (2%)	0 (0%)	5 (3%)	0 (0%)
Other***	35 (11%)	9 (10%)	14 (9%)	12 (15%)
Prefer not to say	1 (<1%)	0 (0%)	0 (0%)	1 (1%)
Level of formal education				
Primary/intermediate school	7 (2%)	6 (7%)	1 (1%)	0 (0%)
College/high school	59 (18%)	23 (26%)	35 (22%)	1 (1%)
Undergraduate	102 (31%)	25 (29%)	51 (32%)	26 (33%)
Postgraduate	102 (31%)	16 (18%)	36 (23%)	50 (63%)
Trade/technical/vocational training	41 (13%)	14 (16%)	26 (16%)	1 (1%)
Other	7 (2%)	2 (2%)	3 (2%)	2 (3%)
Prefer not to say	8 (2%)	1 (1%)	7 (4%)	0 (0%)

NZ: New Zealand.

* All of the participant roles/identities rather than the identity/role they chose for completion of the survey.

** Includes additional non-Māori ethnicities for some participants.

*** Includes African nations, North and South American, European and a combination of ethnicities.

### Perceptions of early support

Table 2 details participant perceptions of early support including the results of the Chi-square analyses. Compared to parents and professionals, a significantly greater proportion of autistic adults indicated that the appropriateness of early support ‘depended on the nature of those supports’ and a smaller proportion indicated that early support was ‘appropriate’. A few participants stated that it was not appropriate to provide early support. Of those who believed early support was ‘appropriate’, the majority agreed with each stated purpose, aside from *reducing characteristics of autism*. The most common models of support across groups were the strengths/difficulties model and the biopsychosocial model.

**Table 2.** Perspectives on early support across autistic adults (*n* = 87), parents (*n* = 159) and professionals (*n* = 80).

Perspective	All *n* (%)	Autistic adults *n* (%)	Parents *n* (%)	Professionals *n* (%)	Missing (*n*)	*p*
Appropriateness					3	<0.001
Appropriate	209 (65%)	32 (38%)*	114 (72%)	63 (79%)		
Not appropriate	4 (1%)	1 (1%)	3 (2%)	0		
It depends	110 (34%)	52 (61%)**	41 (26%)	17 (21%)		
Purpose^a^					N/A	N/A
New child skills	205 (98%)	30 (97%)	114 (100%)	61 (97%)		
Removing barriers	182 (87%)	28 (90%)	98 (86%)	56 (89%)		
Reducing autism characteristics	63 (30%)	4 (13%)	34 (30%)	25 (40%)		
Improving child mental health/well-being	182 (87%)	28 (90%)	100 (88%)	54 (86%)		
Improving child and family quality of life	186 (89%)	28 (90%)	100 (88%)	58 (92%)		
Increasing child autonomy	150 (72%)	22 (71%)	75 (66%)	53 (84%)		
Increasing child advocacy	148 (71%)	24 (77%)	80 (70%)	46 (73%)		
Model of support					8	<0.001
Medical	17 (5%)	3 (4%)	10 (6%)	4 (5%)		
Social	51 (15%)	24 (30%)**	19 (12%)	8 (10%)		
Biopsychosocial	114 (36%)	13 (16%)*	47 (30%)	54 (71%)**		
Strengths/difficulties	126 (40%)	39 (48%)	75 (48%)	12 (17%)*		
Other	7 (2%)	2 (2%)	4 (3%)	1 (1%)		

N/A: not applicable.

a Completed for participants who stated that early support was ‘appropriate’.

* A significantly lower proportion of participants in this group aligned with this perspective compared to other participant groups (adjusted *p* ⩽ 0.05).

** A significantly higher proportion of participants in this group aligned with this perspective compared to other participant groups (adjusted *p* ⩽ 0.05).

#### Associations between participant group and perceptions of early support

A significantly greater proportion of autistic adults aligned with a social model compared to both parents and professionals. A significantly greater proportion of professionals aligned with a biopsychosocial model compared to both parents and autistic adults, and the proportion of parents aligning with this model was greater than the proportion of autistic adults. A significantly greater proportion of autistic adults and parents aligned with a strengths/difficulties model compared to professionals.

### Support goals priorities

Table 3 indicates the mean priority score and ranking for each goal domain across participant groups, and Supplementary Table S3 provides the priority scores for each individual goal. Across participants, the highest priorities were unanimously *adult supports*, followed by either *reducing harmful behaviour* or *quality of life*. The overall lowest priorities were *play skills* and *autism characteristics*.

**Table 3.** Goal priority scores across domains (*n* = 311).

Goal domain	Group								Missing (*n*)
	All		Autistic adults		Parents		Professionals		
	M (SD)	Rank	M (SD)	Rank	M (SD)	Rank	M (SD)	Rank	
Adult supports	1.11 (0.20)	1	1.08 (0.20)	1	1.11 (0.22)	1	1.12 (0.18)	1	35
Reducing harmful behaviour	1.16 (0.50)	2	1.13 (0.51)	3	1.15 (0.39)	2	1.27 (0.71)	2	29
Quality of life	1.20 (0.34)	3	1.11 (0.17)	2	1.21 (0.29)	3	1.28 (0.51)	3	35
Communication	1.89 (0.61)	4	2.03 (0.68)	4	1.83 (0.59)	4	1.87 (0.57)	5	16
Daily living skills	2.01 (0.72)	5	2.04 (0.76)	5	2.04 (0.71)	5	1.93 (0.70)	6	33
Participation	2.19 (0.87)	6	2.57 (0.94)	7	2.20 (0.84)	6	1.82 (0.69)	4	34
Motor skills	2.27 (0.78)	7	2.36 (0.86)	6	2.23 (0.75)	7	2.27 (0.75)	7	30
Academic skills	2.64 (0.92)	8	2.58 (0.99)	8	2.53 (0.89)	8	2.90 (0.86)	10	30
Play skills	2.65 (0.79)	9	3.02 (0.81)	9	2.54 (0.71)	9	2.49 (0.80)	9	39
Autism characteristics	2.69 (0.80)	10	3.22 (0.78)	10	2.56 (0.73)	10	2.42 (0.72)	8	50

SD: standard deviation.

Possible scores ranged from 1 (high priority) to 4 (not at all a priority/not appropriate). The rank column indicates the order of priority scores from highest to lowest priority across all participants within each group.

#### Associations between overall demographic characteristics, perspectives on early supports and support goal priorities

Supplementary Table S7 shows the results of the hierarchical linear regressions examining overall demographic characteristics and perspectives on early supports as predictors of support goal priorities in each examined domain except *adult supports, reducing harmful behaviours* or *quality of life* due to limited variation. In all models, the combined variables within both the demographic characteristics and perspectives on early support blocks were significant predictors of mean priority scores, except *motor skills* and *daily living skills* for which combined demographic characteristics were not significant predictors. The cumulative *R*^2^ values ranged from 0.177 for *motor skills* and 0.433 for *autism characteristics* indicating that these models explained 17.7%–43.3% of the total variation in mean priority scores.

Regarding demographic characteristics, participant primary identity/role was a significant predictor for the *autism characteristics, play skills* and *participation* domains. The mean priority scores for *autism characteristics*, *participation* and *play skills* were higher for professionals compared to autistic adults. The level of formal education was a significant predictor in the *academic skills* and *play skills* goal domains. Compared to participants with at least some high school education, participants with postgraduate education rated *academic* and *play skills* as a lower priority. Country of residence, gender, ethnicity and experience with young children were not significant predictors in any of the models.

Across all examined domains, except motor skills, participants who stated that the appropriateness of early supports ‘depended on the nature of those supports’ had lower mean goal priority scores than those who stated that early support was appropriate. Those who aligned with a social model also had lower mean priority scores in all goal domains except *communication* and *academic skills*, compared to those who aligned with a medical model, as did those who aligned with a biopsychosocial model for the *play skills* domain only.

#### Associations between unique demographic characteristics and support goal priorities

Supplementary Tables S8–S10 show the results of the multiple regressions examining demographic characteristics unique to each participant group as predictors of support goal priorities in each domain except *adult supports*, *reducing harmful behaviours* or *quality of life* due to limited variation. The combined autistic adult, parent and professional unique demographic characteristics were not significant predictors of mean priority scores in any domain. However, parents whose children were diagnosed with global developmental delay (GDD) rated goals in the *play* and *daily living skills* domains as a significantly higher priority than parents whose children were not diagnosed with GDD. The remaining parent demographic characteristics were not significant predictors.

### Appropriateness of support goals

Table 4 shows the number of participants who rated at least one goal within each domain as inappropriate and the results of the Chi-square analyses, while Supplementary Table S3 shows those who rated each specific goal as inappropriate. Across all participant groups, the fewest participants rated goals within *adult supports* and *reducing harmful behaviour* as inappropriate, while more than a quarter of participants rated at least one goal within the *play skills* domain as inappropriate, and almost half of participants found at least one goal within the *autism characteristics* domain to be inappropriate. Specific goals regarding the child making eye contact and reducing ‘intense’ interests and ‘restricted and repetitive behaviours’ within the *autism characteristics* domain were rated as inappropriate by around a third of participants.

**Table 4.** The number and percentage of participants who rated at least one goal within each domain as ‘inappropriate’ (*n* = 311).

Goal domain	All	Autistic adults	Parents	Professionals	Missing (*n*)	*p*
Adult supports	1 (<1%)	1 (<1%)	0	0	32	N/A
Reducing harmful behaviours	5 (2%)	0	2 (1%)	3 (1%)	27	N/A
Quality of life	13 (4%)	1 (<1%)	5 (2%)	7 (2%)	28	N/A
Motor skills	19 (7%)	7 (9%)	8 (6%)	4 (5%)	20	0.494
Daily living skills	24 (8%)	8 (11%)	11 (8%)	3 (4%)	22	0.289
Communication	28 (9%)	11 (14%)	11 (7%)	6 (8%)	0	0.208
Participation	28 (10%)	**16 (22%)** *	13 (9%)	3 (4%)	23	<0.001
Academic skills	29 (10%)	6 (8%)	12 (8%)	11 (14%)	18	0.298
Play skills	76 (26%)	**35 (47%)** *	31 (22%)	10 (14%)	22	<0.001
Autism characteristics	128 (44%)	**55 (72%)** *	49 (35%)	24 (36%)	18	<0.001

N/A: not applicable.

To control for type 1 error across analyses, significance was set at *p* ⩽ 0.007.

* A higher proportion of participants in this group rated at least one goal in this domain as inappropriate compared to other participant groups (adjusted *p* ⩽  0.05).

#### Associations between participant group and the appropriateness of support goals

A significantly greater proportion of autistic adults found at least one goal in each of the *autism characteristics, play skills* and *participation* domains to be inappropriate compared with parents and professionals. There were no differences between groups for the remaining examined goal domains.

## Discussion

In this study, the highest priority support goals across participant groups related to adults changing/modifying their approach to better support the child, the reduction and replacement of behaviours which cause harm to the child and others and improving child quality of life. These goals are consistent with the neurodiversity movement because of their focus on improving the child’s well-being by changing the physical and social environment rather than the child (Gillespie-Lynch et al., 2017; Pellicano & den Houting, 2022). In contrast to our hypotheses, our results suggest that both autistic and non-autistic participants in this study prioritised neurodiversity-affirming goals. It is noteworthy that the autistic and autism communities in New Zealand and Australia identified the same goals as the highest priority for young autistic children, as other research and commentaries often highlight disagreements among these communities (Kenny et al., 2016; Leaf et al., 2022; Schuck et al., 2022).

In line with previous research, goals related to communication and daily living skills were also a high priority for all participants groups in this study (Ghanadzade et al., 2018; Kapp et al., 2013; Pituch et al., 2011; Rodger et al., 2004). These goals focus on providing the child with a meaningful way of advocating for themselves and their needs and increasing their self-determination (Leadbitter et al., 2021). Indeed, autistic individuals have advocated for providing autistic children with ‘an effective and robust method of communication as the first priority’ during early support (ASAN, 2021, p. 22) and specify that this may include augmentative and alternative communication depending on the preferences of the child.

While participants in this study generally agreed that goals related to autism characteristics were a lower priority, autistic adults rated these goals even lower and as more likely to be inappropriate than parents and/or professionals. Many autistic adults are strong supporters and proponents of the neurodiversity movement and view their autism as an inseparable part of their identity (Kapp et al., 2013; Pellicano & den Houting, 2022). Thus, goals targeting autism characteristics can be seen as trying to change the child’s core identity and even attempting to cure autism. While parents and professionals were evidently aware of the challenges surrounding these types of goals, including the risk of encouraging a child to ‘mask’ their autistic characteristics (Miller et al., 2021), it is possible that this was not as salient for these groups compared to autistic adults. Autistic adults also rated goals targeting participation and play skills as a lower priority than professionals, and as more likely to be inappropriate than both parents and professionals. This may be for similar reasons. That is, many autistic adults advocate that there is no one ‘correct’ way to play, and it is not necessary for autistic children to conform to how non-autistic children may play (ASAN, 2021; Milton et al., 2012). Participation goals could be also perceived as requiring an autistic child to ‘conform’ or ‘perform’ across settings (ASAN, 2021; Milton, 2012).

Across groups, few participants reported alignment with a medical model of support. This contrasts with Kapp et al. (2013) who reported that non-autistic participants were more likely to endorse seeking a ‘cure’ for autism, one aspect of a medical model perspective. This further indicates a shift in understandings of autism in the decade since Kapp et al.’s study and/or differences between the countries in which the studies took place (New Zealand and Australia vs the United States and the United Kingdom). Although a minority, those who aligned with a medical model also rated goals in the autism characteristics, play skills, daily living skills and participation domains as a higher priority than those who aligned with a social model. This supports the notion that a medical model perspective may be linked to a focus on approaches aimed at prioritising child goals or ‘changing’ the child (Bottema-Beutel et al., 2021; Kapp et al., 2013).

Autistic adults were more likely to align with a social model of support than parents and professionals, and both autistic adults and parents were more likely to align with a model based on the child’s unique strengths and difficulties than professionals. Both of these models fit most closely with neurodiversity principles (Kapp et al., 2013; Pellicano & den Houting, 2022). Professionals were most likely to endorse a biopsychosocial model that focuses on targeting child skills and changing the environment around the child, which may align with the neurodiversity movement depending on the nature of the child skills which are targeted and how they are targeted. Greater alignment with the biopsychosocial model on the part of professionals could be due to their training and the emphasis placed on this approach in published research and by grant funders (Dawson et al., 2022; den Houting et al., 2021). Indeed, the World Health Organization (2001) appears to promote a biopsychosocial approach by advocating for supports for individuals with disabilities to targeted at both the ‘functioning’ of individuals themselves and environmental factors.

Demographic factors that were unique to each participant group did not generally influence goal priority ratings, although parents of children with a diagnosis of GDD rated daily living skills and play skills as a higher priority than parents whose children did not have this diagnosis. By definition, children with this diagnosis experience delays across several developmental domains, and thus, their parents and/or the professionals who support them could be more focused on providing support in these aforementioned areas. However, more research is needed to understand the reasons for this effect, and whether it would apply to non-autistic children with a GDD diagnosis.

Although the study focussed on common support goals for young autistic children, participants were also asked about their perceptions of the appropriateness of providing early support to autistic children in general. The vast majority of participants stated that it was either appropriate to provide early support or that it could be appropriate, depending on the nature of those supports. Autistic adults were also more likely than parents or professionals to express caution around the appropriateness of all early supports. This caution predicted lower mean priority ratings across most child goal domains. Qualitative comments (to be analysed separately) indicated that participants across all groups expressed concern about the use of supports, predominantly based on the principles of applied behaviour analysis, which have resulted in harm to autistic people and include goals focused on reducing characteristics of autism, ‘normalising’ the child and/or making them ‘indistinguishable from their peers’ (ASAN, 2021; Leaf et al., 2022).

There are several implications of these findings. First, all participant groups agreed on the highest priority goals, which suggests that the autistic and autism communities in New Zealand and Australia are placing increasing importance on neurodiversity-affirming goals for young autistic children, and on listening to the views of the autistic community. Importantly, many professionals and parents appear to be aware of the potential inappropriateness of goals targeting eye contact and ‘restricted and repetitive’ behaviours, but they may be less aware that certain participation and play goals could also be associated with attempts to ‘normalise’ autistic children.

This study was limited in several ways. First, as autistic children were not eligible to participate, it is not possible to determine the priorities of the children themselves in relation to support goals. Although we endeavoured to make the survey accessible for all, improving the accessibility of the study as a whole may have captured a broader range of participants. Furthermore, few autistic adults who participated were themselves diagnosed with an intellectual disability or had received support services when they were young. The average age of diagnosis was also 27 years. Thus, the results may not represent the full range of autistic adult perspectives. Twenty-nine participants also did not complete the full survey, which indicates that it may have been too long or onerous for some individuals. In this survey, we used short, lay descriptions of complex models of support (e.g. the medical and social models). These descriptions did not cover all nuances of these models, and it is possible that participants would have responded differently if they had more detail or understanding of these theoretical approaches. The specific items chosen for each goal domain also only represent a small proportion of all possible goals within these categories. Furthermore, while Māori, the Indigenous People of New Zealand, were quite well-represented in the survey relative to the population, Aboriginal and Torres Strait Islanders were not. While the study included goals aimed at upskilling the adults around the child, it did not evaluate goals aimed at changing the broader environment, such as creating inclusive education practices, or improving the quality of life and well-being of the wider family. While all participants were instructed to rate goals in relation to a hypothetical child, it is likely that they were also influenced by their own varied lived, familial and/or professional experience of autism and their experience with young children more generally. Furthermore, determinations of the appropriateness and importance of goals for a hypothetical 3-year-old who was presumed to be ‘developmentally capable’ of each goal may not reflect participants’ perceptions of support goals for young autistic children more broadly. Finally, self-report responses are subject to biases; thus, the participants’ prioritisation of goals in reality may differ from their statements in this survey.

More research is also needed to understand the reasons why participants rated certain goals as lower or higher priority or inappropriate. Another important avenue of research relates to goal priorities for older autistic children and adults, and an examination of the way goal priorities may change over time. Research is also needed to understand autistic children’s goal priorities for themselves, as well as the perceptions of autistic adults who were diagnosed early and accessed early support. This will likely require various research methods, tailored to the needs of the individual. Future research could also focus exclusively on the perceptions of parents who have recent experiences of early support for their autistic children. Although goals targeted at autism characteristics were among the lowest priorities across groups, many large clinical studies continue to assess reductions on measures of autism characteristics, often as the primary outcome measure. Considerable work needs to be done to align the intentions, goals and outcome measures used in autism research with the priorities of the autistic and autism communities. In particular, there is a need for a greater understanding of what quality of life looks like for autistic children, and for more accurate and reliable measures of child and family quality of life.

## Supplemental Material

sj-docx-1-aut-10.1177_13623613231168920 – Supplemental material for Community perspectives on the appropriateness and importance of support goals for young autistic children Click here for additional data file. Supplemental material, sj-docx-1-aut-10.1177_13623613231168920 for Community perspectives on the appropriateness and importance of support goals for young autistic children by Hannah Waddington, Hannah Minnell, Lee Patrick, Larah van Der Meer, Ruth Monk, Lisa Woods and Andrew JO Whitehouse in Autism

## References

[bibr1-13623613231168920] American Psychiatric Association. (2013). Diagnostic and statistical manual of mental disorders (5th ed.). 10.1176/appi.books.9780890425596

[bibr2-13623613231168920] Autistic Self Advocacy Network. (2021). For whose benefit? Evidence, ethics, and effectiveness of autism interventions. https://autisticadvocacy.org/policy/briefs/intervention-ethics/

[bibr3-13623613231168920] Bottema-Beutel K. Kapp S. K. Lester J. N. Sasson N. J. Hand B. N. (2021). Avoiding ableist language: Suggestions for autism researchers. Autism in Adulthood, 3(1), 18–29. 10.1089/aut.2020.0014 36601265 PMC8992888

[bibr4-13623613231168920] Carr M. May H . (2008). Te Whāriki: Curriculum voices. In Penn H. (Ed.), Early childhood services: Theory, policy and practice (pp. 53–73). Open University Press.

[bibr5-13623613231168920] Cormack D. Robson C. (2010). Classification and output of multiple ethnicities: Considerations for monitoring Māori health. Te Rōpū Rangahau Hauora a Eru Pōmare.

[bibr6-13623613231168920] Dawson G. Franz L. Brandsen S. (2022). At a crossroads – Reconsidering the goals of autism early behavioral intervention from a neurodiversity perspective. JAMA Pediatrics, 176, 839–840. 10.1001/jamapediatrics.2022.2299 35816341 PMC10069446

[bibr7-13623613231168920] den Houting J. Higgins J. Isaacs K. Mahony J. Pellicano E . (2021). ‘I’m not just a guinea pig’: Academic and community perceptions of participatory autism research. Autism, 25(1), 148–163. 10.1177/1362361320951696 32854511

[bibr8-13623613231168920] Engel G. L. (1979). The biopsychosocial model and the education of health professionals. General Hospital Psychiatry, 1(2), 156–165. 10.1016/0163-8343(79)90062-8 499786

[bibr9-13623613231168920] Ghanadzade M. Waltz M. Ragi T. (2018). The intervention priorities of parents of children with autism spectrum disorders in Iran. Research in Autism Spectrum Disorders, 55, 14–24. 10.1016/j.rasd.2018.08.002

[bibr10-13623613231168920] Gillespie-Lynch K. Kapp S. K. Brooks P. J. Pickens J. Schwartzman B. (2017). Whose expertise is it? Evidence for autistic adults as critical autism experts. Frontiers in Psychology, 8, Article 438. 10.3389/fpsyg.2017.00438 PMC5368186 28400742

[bibr11-13623613231168920] Jonkman K. M. Back E. Begeer S. (2023). Predicting intervention use in autistic children: Demographic and autism-specific characteristics. Autism, 27(2), 428–442. 35695079 10.1177/13623613221102748

[bibr12-13623613231168920] Kapp S. K. Gillespie-Lynch K. Sherman L. E. Hutman T. (2013). Deficit, difference, or both? Autism and neurodiversity. Developmental Psychology, 49(1), 59–71. 10.1037/a0028353 22545843

[bibr13-13623613231168920] Kenny L. Hattersley C. Molins B. Buckley C. Povey C. Pellicano E. (2016). Which terms should be used to describe autism? Perspectives from the UK autism community. Autism, 20(4), 442–462. 10.1177/1362361315588200 26134030

[bibr14-13623613231168920] Leadbitter K. Buckle K. L. Ellis C. Dekker M. (2021). Autistic self-advocacy and the neurodiversity movement: Implications for autism early intervention research and practice. Frontiers in Psychology, 12, Article 782. 10.3389/fpsyg.2021.635690 PMC8075160 33912110

[bibr15-13623613231168920] Leaf J. B. Cihon J. H. Leaf R. McEachin J. Liu N. Russell N. . . . Khosrowshahi D. (2022). Concerns about ABA-based intervention: An evaluation and recommendations. Journal of Autism and Developmental Disorders, 52(6), 2838–2853. 10.1007/s10803-021-05137-y 34132968 PMC9114057

[bibr16-13623613231168920] Miller D. Rees J. Pearson A. (2021). ‘Masking is life’: Experiences of masking in autistic and non-autistic adults. Autism in Adulthood, 3(4), 330–338. 10.31219/osf.io/t6vp2 36601640 PMC8992921

[bibr17-13623613231168920] Milton D. E. (2012). On the ontological status of autism: The ‘double empathy problem’. Disability & Society, 27(6), 883–887. 10.1080/09687599.2012.710008

[bibr18-13623613231168920] Monk R. Whitehouse A. J. Waddington H. (2022). The use of language in autism research. Trends in Neurosciences, 4, 5791–5793. 10.1016/j.tins.2022.08.009 36184384

[bibr19-13623613231168920] Mullen E. M. (1995). Mullen scales of early learning. American Guidance Service.

[bibr20-13623613231168920] Pellicano E. den Houting J. (2022). Annual Research Review: Shifting from ‘normal science’ to neurodiversity in autism science. Journal of Child Psychology and Psychiatry, 63(4), 381–396. 10.1111/jcpp.13534 34730840 PMC9298391

[bibr21-13623613231168920] Pituch K. A. Green V. A. Didden R. Lang R. O’Reilly M. F. Lancioni G. E. Sigafoos J. (2011). Parent reported treatment priorities for children with autism spectrum disorders. Research in Autism Spectrum Disorders, 5(1), 135–143. 10.1016/j.rasd.2010.03.003

[bibr22-13623613231168920] Rochford T. (2004). Whare Tapa Whā: A Māori model of a unified theory of health. Journal of Primary Prevention, 25(1), 41–57.

[bibr23-13623613231168920] Rodger S. Braithwaite M. Keen D. (2004). Early intervention for children with autism: Parental priorities. Australasian Journal of Early Childhood, 29(3), 34–41. 10.1177/183693910402900306

[bibr24-13623613231168920] Rogers S. J. Dawson G. (2010). Early start Denver model for young children with autism: Promoting language, learning, and engagement. Guilford Press.

[bibr25-13623613231168920] Schuck R. K. Tagavi D. M. Baiden K. M. Dwyer P. Williams Z. J. Osuna A. . . .Vernon T. W. (2022). Neurodiversity and autism intervention: Reconciling perspectives through a naturalistic developmental behavioral intervention framework. Journal of Autism and Developmental Disorders, 52, 4625–4645. 10.1007/s10803-021-05316-x 34643863 PMC9508016

[bibr26-13623613231168920] Sparrow S. S. Cicchetti D. V. Saulnier C. A. (2016). Vineland-3: Vineland Adaptive Behavior Scales. American Guidance Service.

[bibr27-13623613231168920] VanVoorhis C. W. Morgan B. L. (2007). Understanding power and rules of thumb for determining sample sizes. Tutorials in Quantitative Methods for Psychology, 3(2), 43–50.

[bibr28-13623613231168920] World Health Organization. (2001). International classification of functioning, disability and health: ICF. https://apps.who.int/iris/handle/10665/4240

