# Supplementary material for: Community perspectives on the appropriateness and importance of support goals for young autistic children
Source: Autism. 2023 May 18;28(2):316–26. doi: 10.1177/13623613231168920 (PMC10851621; doi:10.1177/13623613231168920)
Supplement: sj-docx-1-aut-10.1177_13623613231168920 – Supplemental material for Community perspectives on the appropriateness and importance of support goals for young autistic children [file sj-docx-1-aut-10.1177_13623613231168920.docx]

Supplementary Document 1

Survey

**Eligibility Questions**

**Q 1: Which country do you currently live in?**

- New Zealand (continue)
- Australia (continue)
- Other (discontinue message)

**Q 2: Please all that apply:**

- I am a parent/caregiver of a child under 18 years on the autism spectrum (continue)
- I am a professional working with children under 6 years on the autism spectrum (continue)
- I am an autistic adult over the age of 18 (continue)
- None of the above apply (discontinue message)

**Discontinue message:**

Thank you very much for your interest in this study. At this time, we are specifically looking for parents of children under 18 years on the autism spectrum, professionals working with children under 6 years on the autism spectrum, and autistic adults living in New Zealand or Australia. Unfortunately, the answers you provided suggest that you do not meet our current criteria for participation. We appreciate and thank you for your time.

**Section 1: Demographics**

**Questions for all participants**

**Q 1: What is your gender?**

- Male
- Female
- Non- binary
- Prefer not to say
- Other (please specify) __________

**Q 2: Which ethnic group(s) do you belong to? Please select all which apply to you**

- - New Zealand European
  - European Australian
  - Māori
  - Aboriginal or Torres Strait Islander
  - Samoan
  - Cook Islands Māori
  - Tongan
  - Niuen
  - Chinese
  - Indian
  - Vietnamese
  - Other e.g., Japanese, Tokelauan (Please state):___________________
  - Prefer not to say

**Q 3: What is the highest level of education you have completed?**

| - - Primary/Intermediate School   - College/High School   - Trade/technical/vocational training   - Bachelor’s/Undergraduate University Degree | - - Postgraduate University Degree   - Other   (Please describe): _______________   - - Prefer not to say |
| --- | --- |

**Q 4: How much experience do you have with preschool children (0-5 years) who are NOT on the autism spectrum?**

- A lot (I am around them most days)
- Some
- A little
- Very little (I rarely interact with them)

**Q 5: We understand that you may belong to more than one of the following groups, however for this survey please select one identity/role you would like to use:**

- Parent/caregiver
- Professional
- Autistic adult

**Parent/Caregiver:**

**Q 1: What is your relationship to the child?**

- Biological mother
- Biological father
- Stepmother
- Stepfather
- Grandmother
- Grandfather
- Other (Please state): ____________

**Q 2: What is your child’s gender?**

- Male
- Female
- Non-binary
- Prefer not to say
- Other (please specify) __________

**Q 3: How old is your child?**

Years_____

**Q 4: How old was your child when they were diagnosed as being on the autism spectrum?**

Years_________

Months _______

- I don’t know/ prefer not to say

**Q 5: Does your child have any of the following clinical diagnoses in addition to autism?
 Please select all which apply to your child**

| - Attention deficit hyperactivity disorder or attention deficit disorder - Global developmental delay - Anxiety - Depression | - Intellectual disability - Other; Please state:___________ - No other diagnoses - I prefer not to say |
| --- | --- |

**Q 6: Which ethnic group(s) does your child belong to?**

**Please select all which apply to your child**

| - - New Zealand European   - European Australian   - Māori   - Aboriginal or Torres Strait Islander   - Samoan   - Cook Islands Māori   - Tongan | - - Niuean   - Chinese   - Indian   - Vietnamese   - Other e.g., Japanese, Tokelauan (please state):___________________   - Prefer not to say |
| --- | --- |

**Q 7: Does your child currently receive any professional support services*?**

**Professional support services may also be referred to as intervention or therapy. For the purposes of this study professional support services do not include respite care or support from educators.*

- Yes (please select all that apply):
- Speech and language therapy
- Occupational therapy
- ABA-based therapy
- Psychologist support
- Drama therapy
- Play therapy
- Art therapy
- Music therapy
- Physiotherapy
- Relationship-based therapy
- Social skills support
- Cognitive behavioural therapy (CBT)
- Parent education/training programmes
- Behaviour support
- Multidisciplinary interventions
- Other (please state): _________________
- No, my child does not currently receive professional supports

**Q 8: How many hours of support does your child receive on average across all services per month?**

**_________________** hours

**Q 9: Did your child receive professional supports when they were preschool aged (0-5 years)?**

- Yes (please select all that apply):
- Physiotherapy
- Relationship-based therapy
- Social skills support
- Cognitive behavioural therapy (CBT)
- Parent education/training programmes
- Behaviour support
- Multidisciplinary interventions
- Other (please state): _________________
- Speech and language therapy
- Occupational therapy
- ABA-based therapy
- Psychologist support
- Drama therapy
- Play therapy
- Art therapy
- Music therapy
- My child is currently preschool aged, so their professional supports are listed above.
- No, my child did not receive professional support when they were preschool aged/My child is currently preschool aged and has not received any supports

[If participants answered yes] **How many hours of professional support services did your child receive across all services per month?**

**_________________** hours

**Professionals:**

**Q 1: What is your current role?**

- Play therapist
- Art therapist
- Music therapist
- Social Worker
- Physiotherapist
- Board certified behaviour analyst
- Behaviour therapist (not board certified)

Other (Please specify) ______________

- General Practitioner
- Nurse
- Paediatrician
- Psychiatrist
- Psychologist
- Occupational Therapist
- Speech Pathologist
- Drama therapist

**Q 2: How long have you been in this role?**

Years___________

**Q 3: How many years of experience do you have working in clinical practice with children on the autism spectrum?**

Years___________

**Autistic Adults:**

**Q 1: How old are you?**

- 18-25
- 26-30
- 31-40
- 41-50
- Over 50
- Prefer not to say

**Q 2: How old were you when you were diagnosed as being on the autism spectrum?**

Years _________

Months _______

- I don’t know/ prefer not to say

**Q 3: Have you received any of the following clinical diagnoses in addition to autism?
 Please select all which apply**

| - Attention deficit hyperactivity disorder or attention deficit disorder - Global developmental delay - Anxiety - Depression | - Intellectual disability - Other (Please state):___________ - No other diagnoses - I prefer not to say |
| --- | --- |

**Q 4: Did you receive autism-specific professional support services when you were preschool aged (0 – 5 years)?**

- Yes (Please select all that apply):
- Physiotherapy
- Relationship-based therapy
- Social skills support
- Cognitive behavioural therapy (CBT)
- Parent education/training programmes
- Behaviour support
- Multidisciplinary interventions
- Other (please state): _________________
- No
- Speech and language therapy
- Occupational therapy
- ABA-based therapy
- Psychologist support
- Drama therapy
- Play therapy
- Art therapy
- Music therapy
- I was not diagnosed at preschool age
- I don’t know
- Prefer not to say
- Optional free text to tell us more about this

**If you answered yes to the above, how many hours of professional support services did you receive across all services per month?**

**_________________** hours

**Section 2: Perspective of early professional supports**

**Q 1: Do you personally believe that it is appropriate to provide early professional support* to pre-school aged children on the autism spectrum?**

**Professional supports may also be referred to as intervention or therapy. This may include, but is not limited to, speech and language therapy, occupational therapy, ABA-based therapy, psychologist support, physical therapy, play therapy, drama therapy, art therapy, music therapy, social skills support, Cognitive behavioural therapy (CBT), parent education/training programs, routine/play-based therapy, behaviour support, multidisciplinary interventions, relationship-based therapy )*

- Yes
- No
- It depends on the nature of those supports
- Don’t know
- Prefer not to say
- Optional free text to tell us more about this

**If you like, you can tell us more about why you selected your response**

**Q 2:** [If participants answered yes to the above] **What do you believe is the purpose of providing these early supports?**

**Please select all which apply to you**

- To help children on the autism spectrum gain skills in areas where they may be experiencing difficulties (e.g. physical, cognitive, communication, social and emotional)
- To help increase the ability of children to function without barriers in their daily life
- To reduce the diagnostic behaviours of autism (e.g. fewer restricted and repetitive behaviours)
- To support positive mental health and wellbeing for the child
- To improve quality of life for the child and family more generally
- To increase the autonomy of children on the autism spectrum
- To provide children on the autism spectrum with the ability to advocate for themselves
- Free-text box

**Q 3: Which of these best aligns with your personal perspective of what early supports should focus on?**

**Please select all which apply**

Early supports should focus on:

- Directly building new skills and reducing impairments within the child
- Making specific changes to the environments around the child, along with broader changes to society
- Aspects of both the child’s development and the environment in which the child is situated
- Helping the child identify their own strengths and difficulties and find their own coping mechanisms.
- None of these align with my perspective of what early supports should focus on: ________________

**Section 3: Goals for early supports**

In this section, there will be a list of goals that are typically in different types of professional support services for children on the autism spectrum. We will be asking you if you think they are appropriate and/or a priority for a young child on the autism spectrum. We understand that you are answering with your own opinion, rather than on behalf of all parents/professionals/autistic adults.

To answer these questions, imagine that each possible goal is something that a 3-year-old child on the autism spectrum is developmentally able to do but is not doing most of the time.

|  | Not an appropriate goal | An appropriate goal and | | | | No opinion/Not sure |
| --- | --- | --- | --- | --- | --- | --- |
|  |  | Not at all a priority | Low priority | Medium priority | High priority |  |
| The child verbally or non-verbally* communicates what they want/need and what they don't want/need in an understandable way |  |  |  |  |  |  |
| The child verbally or non-verbally comments on, labels, and describes the world around them |  |  |  |  |  |  |
| The child verbally or non-verbally expresses their feelings |  |  |  |  |  |  |
| The child verbally or non-verbally asks and answers questions |  |  |  |  |  |  |
| The child understands and follows simple instructions |  |  |  |  |  |  |
| The child points to or shows body parts or common objects when asked |  |  |  |  |  |  |

**Goals:**

*Non-verbally includes any type of augmentative or alterative communication including written communication and use of signing, picture exchange, communication boards, or a speech generating device.
If you like, you can tell us more about why you selected your responses **(this is optional, and not included in the estimated time limit).**

|  | Not an appropriate goal | An appropriate goal and | | | | No opinion/Not sure |
| --- | --- | --- | --- | --- | --- | --- |
|  |  | Not at all a priority | Low priority | Medium priority | High priority |  |
| The child engages in social activities with others (for example, greeting others, playing social games such as peek-a-boo or singing songs) |  |  |  |  |  |  |
| The child attends to another person with eye contact |  |  |  |  |  |  |
| The child makes or tries to make social contact with others (by smiling, reaching, making noises, talking etc) |  |  |  |  |  |  |
| The child imitates movements/ actions/ facial expressions/ sounds performed by another person |  |  |  |  |  |  |

If you like, you can tell us more about why you selected your responses **(this is optional, and not included in the estimated time limit).**

|  | Not an appropriate goal | An appropriate goal and | | | | No opinion/Not sure |
| --- | --- | --- | --- | --- | --- | --- |
|  |  | Not at all a priority | Low priority | Medium priority | High priority |  |
| The child matches letters, numbers, pictures and shapes |  |  |  |  |  |  |
| The child sorts similar objects into categories/ groups (e.g. colour, shape, type of object) |  |  |  |  |  |  |
| The child counts (verbally or non-verbally) numbers up to 5 |  |  |  |  |  |  |
| The child verbally or non-verbally identifies some letters |  |  |  |  |  |  |

If you like, you can tell us more about why you selected your responses **(this is optional, and not included in the estimated time limit).**

|  | Not an appropriate goal | An appropriate goal and | | | | No opinion/Not sure |
| --- | --- | --- | --- | --- | --- | --- |
|  |  | Not at all a priority | Low priority | Medium priority | High priority |  |
| The child walks, runs, climbs and jumps without falling |  |  |  |  |  |  |
| The child stacks objects, puts objects inside other objects and takes them out |  |  |  |  |  |  |
| The child holds a pencil or crayon and uses it to draw lines, scribbles and/or shapes |  |  |  |  |  |  |

If you like, you can tell us more about why you selected your responses **(this is optional, and not included in the estimated time limit).**

|  | Not an appropriate goal | An appropriate goal and | | | | No opinion/Not sure |
| --- | --- | --- | --- | --- | --- | --- |
|  |  | Not at all a priority | Low priority | Medium priority | High priority |  |
| The child plays alone with minimal adult supervision |  |  |  |  |  |  |
| The child plays with others with minimal adult supervision |  |  |  |  |  |  |
| The child shares toys and possessions with others |  |  |  |  |  |  |
| The child uses toys for their intended purpose |  |  |  |  |  |  |
| The child uses toys/objects for make believe play (e.g. pretending a block is a car) |  |  |  |  |  |  |

If you like, you can tell us more about why you selected your responses **(this is optional, and not included in the estimated time limit).**

|  | Not an appropriate goal | An appropriate goal and | | | | No opinion/Not sure |
| --- | --- | --- | --- | --- | --- | --- |
|  |  | Not at all a priority | Low priority | Medium priority | High priority |  |
| The child begins to eat and drink independently |  |  |  |  |  |  |
| The child begins to independently use the toilet/potty |  |  |  |  |  |  |
| The child begins to independently dress and undress themselves |  |  |  |  |  |  |
| The child begins to independently wash their hands and body |  |  |  |  |  |  |
| The child begins to help with household tasks (e.g., tidying up and helping to feed animals) |  |  |  |  |  |  |

If you like, you can tell us more about why you selected your responses **(this is optional, and not included in the estimated time limit).**

|  | Not an appropriate goal | An appropriate goal and | | | | No opinion/Not sure |
| --- | --- | --- | --- | --- | --- | --- |
|  |  | Not at all a priority | Low priority | Medium priority | High priority |  |
| The child actively participates at home (e.g., is involved in family activities such as mealtime by joining in with conversation and interacting with others) |  |  |  |  |  |  |
| The child actively participates in environments outside of home (e.g., in a community playground, going to the supermarket) |  |  |  |  |  |  |
| The child actively participates in activities/routines in an early childhood education setting (e.g., joins in with songs/games at mat time) |  |  |  |  |  |  |

If you like, you can tell us more about why you selected your responses **(this is optional, and not included in the estimated time limit).**

|  | Not an appropriate goal | An appropriate goal and | | | | No opinion/Not sure |
| --- | --- | --- | --- | --- | --- | --- |
|  |  | Not at all a priority | Low priority | Medium priority | High priority |  |
| The child is less attached to specific routines and behaviours (e.g. always needing to drive the same way home, becoming very upset when routines change) |  |  |  |  |  |  |
| The child has less intense and specific interests in objects and/or topics (e.g. numbers, emergency vehicles) |  |  |  |  |  |  |
| The child has less sensory behaviours. This includes over-reactivity (e.g. difficulty coping with light/noise) and under-reactivity (e.g. not seeming to notice when they are cold or in pain) |  |  |  |  |  |  |
| The child shows less restrictive, repetitive behaviours (e.g., flapping their arms or rocking), speech, and play |  |  |  |  |  |  |
| The child stops behaviours that harm others (e.g., hitting and biting) and instead uses non-harmful behaviours which meet the same need |  |  |  |  |  |  |
| The child stops behaviours that harm themselves (e.g., head banging, hand biting) and instead uses non-harmful behaviours which meet the same need. |  |  |  |  |  |  |

If you like, you can tell us more about why you selected your responses **(this is optional, and not included in the estimated time limit).**

|  | Not an appropriate goal | An appropriate goal and | | | | No opinion/Not sure |
| --- | --- | --- | --- | --- | --- | --- |
|  |  | Not at all a priority | Low priority | Medium priority | High priority |  |
| The child has good physical health |  |  |  |  |  |  |
| The child has good mental health |  |  |  |  |  |  |
| The child is safe |  |  |  |  |  |  |
| The child has a caring and supportive family and wider community |  |  |  |  |  |  |
| The child develops a sense of cultural identity/belonging |  |  |  |  |  |  |
| The child is able to pursue their own interests |  |  |  |  |  |  |
| The child has autonomy over their own decisions where possible |  |  |  |  |  |  |

If you like, you can tell us more about why you selected your responses **(this is optional, and not included in the estimated time limit).**

The following section relates to goals for adults supporting the child on the autism spectrum. This could be parents, family members, teachers, other professionals, or members of the community.

|  | Not an appropriate goal | An appropriate goal and | | | | No opinion/Not sure |
| --- | --- | --- | --- | --- | --- | --- |
|  |  | Not at all a priority | Low priority | Medium priority | High priority |  |
| The adult understands and responds appropriately to the child's natural ways of communicating |  |  |  |  |  |  |
| The adult engages in social activities, routines and play with the child in a way that is fun and engaging |  |  |  |  |  |  |
| The adult adapts their routine and environment in a way that allows the child to actively participate |  |  |  |  |  |  |
| The adult enables the child to meet their sensory needs |  |  |  |  |  |  |
| The adult supports the child with changes in routines |  |  |  |  |  |  |
| The adult supports the child to engage in their special interests |  |  |  |  |  |  |

If you like, you can tell us more about why you selected your responses **(this is optional, and not included in the estimated time limit).**

Thank you very much for responding to this survey. We know how valuable your time is and we appreciate you sharing your perceptions with us. Please feel free to share the survey link with any other people you know who may be interested in participating.

Table S2.

*Comparison of demographic characteristics for participants with data in all domains versus participants with missing data in at least one domain.*

| Demographic characteristic | Analysis | Results | Significant | Description |
| --- | --- | --- | --- | --- |
| **Overall** |  |  |  |  |
| Role | Chi Square | X^2^ (2, N = 326) = 4.294, p = 0.117 | No |  |
| Country | Chi Square | X^2^ (1, N = 326) = 0.016, p = 0.901 | No |  |
| Gender | Chi Square | X^2^ (2, N = 326) = 3.408, p = 0.182 | No |  |
| Ethnicity | Chi Square | X^2^ (5, N = 325) = 3.135, p = 0.679 | No |  |
| Level of formal education | Chi Square | X^2^ (6, N = 326) = 17.769, p = 0.007 | Yes | Participants who had missing data in at least one domain were more likely to prefer not to disclose their level of education compared to those who did not have any missing data. |
| Experience with children under 5 | Chi Square | X^2^ (3, N = 326) = 2.513, p = 0.473 | No |  |
|  |  |  |  |  |
| **Autistic adult** |  |  |  |  |
| Age | Chi Square | X^2^ (1, N = 87) = 4.237, p = 0.551 | No |  |
| Age of diagnosis (years) M(SD) | Independent Samples T-test | *t*(151) = -0.452, p = 0.645 | No |  |
| Additional diagnoses |  |  |  |  |
| Anxiety | Chi Square | X^2^ (1, N = 86) = 0.376, p = 0.540 | No |  |
| ADHD/ADD | Chi Square | X^2^ (1, N = 86) = 0.556, p = 0.347 | No |  |
| Depression | Chi Square | X^2^ (1, N = 86) = 0.149, p = 0.699 | No |  |
| Other | Chi Square | X^2^ (1, N = 86) = 1.115, p = 0.291 | No |  |
|  |  |  |  |  |
| **Parent** |  |  |  |  |
| Relationship to child | Chi Square | X^2^ (4, N = 158) = 2.928, p = 0.570 | No |  |
| Child gender | Chi Square | X^2^ (2, N = 159) = 3.930, p = 0.140 | No |  |
| Child age (years) | Mann-Whitney U | U = 2301, p = 0.813 | No |  |
| Child age of diagnosis (years) | Mann-Whitney U | U = 2374, p = 0.630 | No |  |
| Child additional diagnoses |  |  |  |  |
| Anxiety | Chi Square | X^2^ (1, N = 159) = 0.001, p = 0.976 | No |  |
| ADHD/ADD | Chi Square | X^2^ (1, N = 159) = 0.496, p = 0.481 | No |  |
| Depression | Chi Square | X^2^ (1, N = 159) = 0.461, p = 0.497 | No |  |
| Global developmental delay | Chi Square | X^2^ (1, N = 159) = 2.433, p = 0.119 | No |  |
| Intellectual and developmental  disability | Chi Square | X^2^ (1, N = 159) = 0.028, p = 0.867 | No |  |
| Other | Chi Square | X^2^ (1, N = 159) = 0.689, p = 0.407 | No |  |
| Child ethnicity | Chi Square | X^2^ (1, N = 159) = 2.863, p = 0.581 | No |  |
| Currently receiving autism-specific support | Chi Square | X^2^ (1, N = 159) = 2.930, p = 0.087 | No |  |
| Received autism-specific support at 5 years and under | Chi Square | X^2^ (1, N = 159) = 4.691, p = 0.196 | No |  |
|  |  |  |  |  |
| **Professional** |  |  |  |  |
| Professional role | Chi Square | X^2^ (9, N = 80) = 4.352, p = 0.887 | No |  |
| Years in role | Mann-Whitney U | U = 423.00, p = 0.227 | No |  |
| Years of experience autistic children | Mann-Whitney U | U = 422.50, p = 0.654 | No |  |

Table S3.

*Participant appropriateness and importance ratings for each specific goal across autistic adults, parents, and professionals (n = 311).*

| Goal/Goal domain | Not an appropriate goal | | | |  | Not at all a priority | | | |  | Low priority | | | |  | Medium priority | | | |  | High Priority | | | | No op. | Miss.  (n) |
| --- | --- | --- | --- | --- | --- | --- | --- | --- | --- | --- | --- | --- | --- | --- | --- | --- | --- | --- | --- | --- | --- | --- | --- | --- | --- | --- |
|  | All | AA | Par. | Prof. |  | All | AA | Par. | Prof. |  | All | AA | Par. | Prof. |  | All | AA | Par. | Prof. |  | All | AA | Par. | Prof. |  |  |
| Communication |  |  |  |  |  |  |  |  |  |  |  |  |  |  |  |  |  |  |  |  |  |  |  |  |  |  |
| Expresses wants/needs | 1% | 0% | 1% | < 1% |  | 1% | 0% | 1% | < 1% |  | 4% | 4% | 5% | < 1% |  | 15% | 20% | 16% | 9% |  | **77%** | **71%** | **75%** | **86%** | 3% | 0 |
| Comments | 4% | <1% | 4% | 5% |  | 8% | 19% | 4% | 5% |  | 25% | 23% | 25% | 27% |  | **39%** | **29%** | **42%** | **43%** |  | 22% | 22% | 24% | 18% | 3% | 0 |
| Expresses feelings | 2% | 1% | 2% | 3% |  | 2% | 3% | 1% | 1% |  | 7% | 6% | 6% | 10% |  | 27% | 24% | 28% | 30% |  | **59%** | **58%** | **62%** | **56%** | 3% | 0 |
| Asks/answers questions | 5% | 5% | 5% | 4% |  | 4% | 5% | 2% | 5% |  | 23% | 24% | 23% | 20% |  | **40%** | **34%** | **39%** | **46%** |  | 27% | 25% | 29% | 23% | 3% | 0 |
| Follows instructions | 3% | 5% | 1% | 4% |  | 3% | 9% | 2% | 0% |  | 8% | 15% | 5% | 6% |  | 41% | **44%** | 40% | 38% |  | **42%** | 20% | **49%** | **49%** | 3% | 0 |
| Points | 5% | 8% | 2% | 6% |  | 10% | 14% | 8% | 9% |  | **33%** | **29%** | 31% | **39%** |  | 32% | **29%** | **37%** | 23% |  | 18% | 14% | 20% | 20% | 3% | 0 |
| Autism characteristics |  |  |  |  |  |  |  |  |  |  |  |  |  |  |  |  |  |  |  |  |  |  |  |  |  |  |
| Engages in social  activities | 8% | 18% | 4% | 4% |  | 8% | 13% | 7% | 4% |  | 21% | 26% | 25% | 9% |  | **36%** | **27%** | **37%** | **44%** |  | 25% | 14% | 26% | 36% | 2% | 8 |
| Makes eye contact | **31%** | **53%** | 24% | 22% |  | 15% | 17% | 14% | 13% |  | 28% | 17% | **30%** | **35%** |  | 19% | 8% | 26% | 19% |  | 6% | 4% | 5% | 9% | 2% | 8 |
| Makes social contact | 11% | 22% | 7% | 8% |  | 7% | 12% | 8% | 1% |  | 17% | 19% | 18% | 12% |  | **37%** | **29%** | **41%** | 38% |  | 25% | 10% | 24% | **40%** | 3% | 8 |
| Imitates | 17% | **31%** | 13% | 10% |  | 10% | 24% | 7% | 1% |  | 22% | 17% | 27% | 17% |  | **35%** | 15% | **41%** | **43%** |  | 13% | 5% | 9% | 27% | 3% | 8 |
| Fewer specific routines | 12% | 29% | 7% | 5% |  | 6% | 7% | 7% | 4% |  | 24% | **30%** | 22% | 21% |  | **38%** | 23% | **41%** | **45%** |  | 18% | 5% | 22% | 25% | 1% | 27 |
| Less intense interests | **33%** | **62%** | 24% | 22% |  | 14% | 15% | 17% | 10% |  | 27% | 12% | **29%** | **36%** |  | 19% | 5% | 21% | 29% |  | 6% | 4% | 7% | 4% | 1% | 27 |
| Less sensory needs | 22% | **43%** | 14% | 18% |  | 6% | 5% | 8% | 4% |  | 14% | 14% | 11% | 19% |  | **36%** | 25% | **39%** | **41%** |  | 19% | 10% | 25% | 16% | 3% | 27 |
| Less restricted/  repetitive behaviour | **35%** | **63%** | 26% | 25% |  | 10% | 8% | 10% | 10% |  | 26% | 12% | **29%** | **34%** |  | 17% | 10% | 20% | 18% |  | 10% | 4% | 12% | 12% | 2% | 27 |
| Academic skills |  |  |  |  |  |  |  |  |  |  |  |  |  |  |  |  |  |  |  |  |  |  |  |  |  |  |
| Matches letters | 6% | 7% | 4% | 9% |  | 16% | 20% | 13% | 16% |  | 29% | 17% | 27% | **42%** |  | **33%** | **33%** | **40%** | 21% |  | 13% | 15% | 13% | 11% | 3% | 18 |
| Sorts objects | 5% | 7% | 5% | 5% |  | 15% | 13% | 12% | 24% |  | 30% | 24% | 32% | **33%** |  | **35%** | **36%** | **38%** | 28% |  | 11% | 13% | 11% | 9% | 3% | 18 |
| Counts to 5 | 7% | 7% | 7% | 7% |  | 16% | 16% | 12% | 24% |  | **30%** | 21% | 30% | **39%** |  | **30%** | **33%** | **32%** | 22% |  | 13% | 15% | 16% | 7% | 4% | 18 |
| Identifies letters | 9% | 8% | 8% | 11% |  | 16% | 16% | 11% | 25% |  | 27% | 17% | 28% | **33%** |  | **32%** | **36%** | **35%** | 24% |  | 13% | 13% | 16% | 7% | 4% | 18 |
| Motor skills |  |  |  |  |  |  |  |  |  |  |  |  |  |  |  |  |  |  |  |  |  |  |  |  |  |  |
| Walks, runs, climbs | 4% | 7% | 4% | 3% |  | 2% | 4% | 2% | 1% |  | 13% | 9% | 15% | 14% |  | **40%** | **43%** | **40%** | 38% |  | 35% | 28% | 35% | **42%** | 4% | 19 |
| Stacks objects | 4% | 7% | 3% | 5% |  | 10% | 11% | 11% | 8% |  | 30% | 23% | 32% | 34% |  | **39%** | **36%** | **42%** | **36%** |  | 11% | 12% | 10% | 13% | 5% | 19 |
| Holds pencil | 4% | 7% | 4% | 3% |  | 9% | 11% | 8% | 8% |  | 30% | 20% | 28% | **45%** |  | **34%** | **36%** | **37%** | 28% |  | 18% | 15% | 21% | 16% | 4% | 19 |
| Play skills |  |  |  |  |  |  |  |  |  |  |  |  |  |  |  |  |  |  |  |  |  |  |  |  |  |  |
| Plays alone | 11% | 12% | 11% | 9% |  | 8% | 11% | 4% | 12% |  | 28% | 20% | 31% | **31%** |  | **37%** | **31%** | **43%** | **31%** |  | 11% | 16% | 9% | 11% | 6% | 22 |
| Plays with others | 9% | 13% | 9% | 5% |  | 6% | 11% | 5% | 4% |  | 22% | 23% | 19% | 26% |  | **39%** | **31%** | **42%** | **41%** |  | 19% | 12% | 22% | 20% | 5% | 22 |
| Shares toys | 11% | 19% | 8% | 8% |  | 9% | 12% | 9% | 4% |  | 23% | **27%** | 19% | 27% |  | **37%** | 25% | **44%** | **36%** |  | 17% | 8% | 19% | 24% | 3% | 22 |
| Uses toys as intended | 22% | **43%** | 16% | 12% |  | 16% | 23% | 13% | 14% |  | **29%** | 16% | **35%** | **31%** |  | 21% | 11% | 24% | 27% |  | 9% | 3% | 9% | 15% | 3% | 22 |
| Uses pretend play | 18% | **35%** | 14% | 8% |  | 12% | 23% | 10% | 7% |  | 26% | 13% | 30% | 30% |  | **31%** | 16% | **35%** | **38%** |  | 10% | 7% | 9% | 18% | 3% | 22 |
| Daily living skills |  |  |  |  |  |  |  |  |  |  |  |  |  |  |  |  |  |  |  |  |  |  |  |  |  |  |
| Eats/drinks  independently | 2% | 4% | 2% | 1% |  | 1% | 0% | 1% | 3% |  | 5% | 5% | 6% | 3% |  | 32% | **44%** | 27% | 28% |  | **56%** | 39% | **61%** | **64%** | 3% | 22 |
| Toilets independently | 4% | 4% | 6% | 1% |  | 2% | 3% | 2% | 3% |  | 13% | 11% | 15% | 12% |  | 35% | 37% | 31% | 41% |  | **42%** | **39%** | **44%** | **42%** | **3%** | 22 |
| Dresses independently | 3% | 3% | 4% | 3% |  | 2% | 4% | 2% | 1% |  | 22% | 23% | 25% | 16% |  | **42%** | **37%** | **42%** | **47%** |  | 27% | 27% | 25% | 31% | 3% | 22 |
| Washes independently | 3% | 3% | 3% | 3% |  | 3% | 3% | 4% | 1% |  | 18% | 21% | 17% | 18% |  | **41%** | **39%** | **42%** | **41%** |  | 33% | 28% | 33% | 36% | 3% | 22 |
| Helps with tasks | 6% | 8% | 7% | 3% |  | 12% | 13% | 12% | 11% |  | **36%** | 20% | **39%** | **44%** |  | 31% | **37%** | 29% | 28% |  | 11% | 13% | 10% | 12^ | 4% | 22 |
| Participation |  |  |  |  |  |  |  |  |  |  |  |  |  |  |  |  |  |  |  |  |  |  |  |  |  |  |
| Participates home | 7% | 16% | 6% | 1% |  | 6% | 5% | 8% | 1% |  | 15% | 11% | 19% | 9% |  | **42%** | **50%** | **36%** | **47%** |  | 26% | 12% | 29% | 36% | 4% | 23 |
| Participates community | 7% | 20% | 4% | 1% |  | 5% | 4% | 2% | 1% |  | 22% | 22% | 25% | 15% |  | **41%** | **35%** | **41%** | **46%** |  | 22% | 14% | 21% | 32% | 4% | 23 |
| Participates ECE | 7% | 18% | 4% | 1% |  | 6% | 8% | 6% | 3% |  | 19% | 22% | 22% | 9% |  | **42%** | **38%** | **42%** | **46%** |  | 23% | 9% | 23% | 36% | 4% | 23 |
| Reducing harmful behaviour |  |  |  |  |  |  |  |  |  |  |  |  |  |  |  |  |  |  |  |  |  |  |  |  |  |  |
| Stops harm others | 1% | 0% | 1% | 4% |  | 1% | 0% | 1% | 0% |  | 0% | 0% | 0% | 0% |  | 9% | 11% | 8% | 8% |  | **89%** | **88%** | **90%** | **88%** | <1% | 27 |
| Stops harm self | 2% | 0% | 1% | 4% |  | <1% | 0% | 1% | 0% |  | 1% | 0% | 2% | 1% |  | 8% | 11% | 9% | 5% |  | **88%** | **86%** | **87%** | **89%** | 1% | 27 |
| Quality of life | 283 | 73 | 138 | 72 |  |  |  |  |  |  |  |  |  |  |  |  |  |  |  |  |  |  |  |  |  |  |
| Good physical health | 2% | 1% | 1% | 6% |  | <1% | 0% | 0% | 1% |  | 1% | 0% | 3% | 1% |  | 16% | 16% | 17% | 13% |  | **80%** | **82%** | **78%** | **79%** | 1% | 28 |
| Good mental health | 1% | 0% | 0% | 4% |  | 0% | 0% | 0% | 0% |  | 1% | 0% | 2% | 1% |  | 5% | 1% | 5% | 8% |  | **92%** | **99%** | **93%** | **85%** | <1% | 28 |
| Is safe | 1% | 0% | 0% | 3% |  | 0% | 0% | 0% | 0% |  | 1% | 0% | 1% | 1% |  | 2% | 1% | 1% | 3% |  | **97%** | **99%** | **98%** | **93%** | 0% | 28 |
| Supportive family | 2% | 0% | 0% | 7% |  | <1% | 1% | 0% | 0% |  | <1% | 0% | 1% | 0% |  | 3% | 1% | 4% | 3% |  | **94%** | **97%** | **95%** | **89%** | <1% | 28 |
| Develops cultural  identity | 1% | 0% | 1% | 3% |  | <1% | 0% | 1% | 0% |  | 7% | 4% | 9% | 6% |  | 23% | 21% | 22% | 25% |  | **68%** | **74%** | **67%** | **65%** | 1% | 28 |
| Pursues own interests | <1% | 0% | 0% | 1% |  | <1% | 0% | 0% | 1% |  | 1% | 0% | 1% | 3% |  | 14% | 8% | 12% | 24% |  | **83%** | **92%** | **86%** | **69%** | 1% | 28 |
| Has autonomy | 1% | 0% | 1% | 3% |  | <1% | 0% | 1% | 0% |  | 4% | 0% | 5% | 6% |  | 20% | 11% | 25% | 21% |  | **74%** | **89%** | **68%** | **69%** | 1% | 28 |
| Adult supports | 279 | 73 | 135 | 71 |  |  |  |  |  |  |  |  |  |  |  |  |  |  |  |  |  |  |  |  |  |  |
| Understands  communication | 0% | 0% | 0% | 0% |  | 0% | 0% | 0% | 0% |  | 0% | 0% | 0% | 0% |  | 4% | 1% | 5% | 3% |  | **96%** | **99%** | **95%** | **97%** | 0% | 32 |
| Adapts social activities | 0% | 0% | 0% | 0% |  | 0% | 0% | 0% | 0% |  | 0% | 0% | 0% | 0% |  | 9% | 5% | 14% | 3% |  | **91%** | **95%** | **85%** | **97%** | 0% | 32 |
| Adapts routine | 0% | 0% | 0% | 0% |  | 0% | 0% | 0% | 0% |  | <1% | 0% | 1% | 0% |  | 15% | 10% | 16% | 18% |  | **85%** | **90%** | **84%** | **82%** | 0% | 32 |
| Supports sensory needs | 0% | 0% | 0% | 0% |  | 0% | 0% | 0% | 0% |  | 1% | 0% | 1% | 1% |  | 9% | 5% | 11% | 8% |  | **90%** | **95%** | **87%** | **90%** | <1% | 32 |
| Supports changes in  routine | <1% | 1% | 0% | 0% |  | 0% | 0% | 0% | 0% |  | <1% | 1% | 0% | 0% |  | 6% | 10% | 5% | 6% |  | **92%** | **88%** | **94%** | **94%** | <1% | 32 |
| Supports interests | 0% | 0% | 0% | 0% |  | 0% | 0% | 0% | 0% |  | 1% | 1% | 0% | 1% |  | 19% | 8% | 21% | 27% |  | **80%** | **90%** | **79%** | **70%** | <1% | 32 |

Note: AA = Autistic adult; Par. = parent; Prof. = professionals; No op. = no opinion/not sure; miss. = missing; ECE = early childhood education. Bold font indicates the most frequent response for each goal and participant group

Table S4.
*Additional demographic characteristics for autistic adults (n= 87)*.

| Demographic Characteristic | N(%)/M(SD) |
| --- | --- |
| Age |  |
| 18 - 25 | 21 (24%) |
| 26 - 30 | 22 (25%) |
| 31 -40 | 20 (23%) |
| 41 -50 | 14 (16%) |
| Over 50 | 9 (10%) |
| Prefer not to say | 1 (1%) |
| Age of diagnosis (years) M(SD) | 27.13 (14.52) |
| Additional diagnoses |  |
| Anxiety | 59 (68%) |
| ADHD/ADD | 38 (44%) |
| Depression | 44 (51%) |
| Global developmental delay | 2 (2%) |
| Intellectual and developmental disability | 3 (3%) |
| Other | 17 (20%) |
| Autism support under 5 years |  |
| Received support | 8 (9%) |
| Did not receive support or cannot recall | 79 (91%) |
| Type of support under 5 year |  |
| ABA therapy | 1 (1%) |
| Art therapy | 2 (2%) |
| Behavioural support | 2 (2%) |
| Cognitive behavioural therapy | 2 (2%) |
| Occupational therapy | 1 (1%) |
| Parent education/training programmes | 1 (1%) |
| Physiotherapy | 1 (1%) |
| Play therapy | 1 (1%) |
| Psychologist support | 1 (1%) |
| Relationship therapy | 1 (1%) |
| Social skills support | 1 (1%) |
| Speech and language therapy | 3 (3%) |
| Other | 2 (2%) |

Table S5.
*Additional demographic characteristics for parents (n = 159).*

| Demographic Characteristic | n(%)/M(SD) |
| --- | --- |
| Relationship to child |  |
| Biological mother | 141 (89%) |
| Biological father | 13 (8%) |
| Grandmother | 2 (1%) |
| Stepfather | 1 (1%) |
| Other (adopted mother, additional guardian) | 2 (1%) |
| Child gender |  |
| Male | 125 (79%) |
| Female | 33 (21%) |
| Gender diverse | 1 (1%) |
| Child age (years) | 8.2 (3.9) |
| Child age of diagnosis (years; | 4.9 (3.2) |
| Child additional diagnoses |  |
| Anxiety | 48 (30%) |
| ADHD/ADD | 41 (26%) |
| Depression | 12 (8%) |
| Global developmental delay | 29 (18%) |
| Intellectual and developmental disability | 11 (7%) |
| Other | 31 (19%) |
| Child ethnicity |  |
| New Zealand/Australian European | 97 (61%) |
| Māori | 36 (23%) |
| Asian | 6 (4%) |
| Pacific Islander | 2 (1%) |
| Other | 18 (11%) |
| Currently receiving autism-specific support |  |
| Yes | 90 (57%) |
| No | 69 (43%) |
| Type of current support |  |
| ABA therapy | 6 (4%) |
| Art therapy | 3 (2%) |
| Behavioural support | 11 (7%) |
| Cognitive behavioural therapy | 2 (1%) |
| Drama therapy | 1 (1%) |
| Multidisciplinary supports | 4 (3%) |
| Music therapy | 12 (8%) |
| Occupational therapy | 47 (30%) |
| Parent education/training programmes | 18 (11%) |
| Physiotherapy | 11 (7%) |
| Play therapy | 9 (6%) |
| Psychologist support | 28 (18%) |
| Social skills support | 16 (10%) |
| Speech and language therapy | 56 (35%) |
| Other | 15 (9%) |
| Received autism-specific support at 5 years and under |  |
| Yes | 94 (59%) |
| No | 62 (39%) |
| Prefer not to say | 3 (2%) |
| Type of autism-specific support at 5 years and younger type |  |
| ABA therapy | 7 (4%) |
| Art therapy | 3 (2%) |
| Behavioural support | 18 (11%) |
| Cognitive behavioural therapy | 1 (1%) |
| Drama therapy | 3 (2%) |
| Multidisciplinary supports | 5 (3%) |
| Music therapy | 6 (4%) |
| Occupational therapy | 45 (28%) |
| Parent education/ training programmes | 25 (16%) |
| Physiotherapy | 9 (6%) |
| Play therapy | 7 (4%) |
| Psychologist support | 13 (8%) |
| Relationship-based therapy | 3 (2%) |
| Social skills support | 14 (9%) |
| Speech and language therapy | 54 (34%) |
| Other | 6 (4%) |

Table S6.
*Additional demographic characteristics for professionals* (n = 80).

| Characteristic | N(%)/ M(SD) |
| --- | --- |
| Professional role |  |
| Behaviour therapist (not board certified) | 7 (9%) |
| Board certified behaviour analyst | 2 (3%) |
| Occupational therapist | 7 (9%) |
| Paediatrician | 7 (9%) |
| Physiotherapist | 1 (1%) |
| Play therapist | 2 (3%) |
| Psychologist | 12 (15%) |
| Social worker | 1 (1%) |
| Speech pathologist | 13 (16%) |
| Other | 28 (35%) |
| Years in role | 10.5 (8.0) |
| Years of experience autistic children | 9.6 (8.0) |

Table S7.

*Hierarchical linear regression predicting mean priority scores across goal domains*

|  | Communication | |  | Autism  characteristics | |  | Academic  skills | |  | Motor skills | |  | Play skills | |  | Daily living skills | |  | Participation | |
| --- | --- | --- | --- | --- | --- | --- | --- | --- | --- | --- | --- | --- | --- | --- | --- | --- | --- | --- | --- | --- |
| Predictors | β | *p* |  | β | *p* |  | β | *p* |  | β | *p* |  | β | *p* |  | β | *p* |  | β | *p* |
| **Demographic characteristics** |  |  |  |  |  |  |  |  |  |  |  |  |  |  |  |  |  |  |  |  |
| Role/Identity |  |  |  |  |  |  |  |  |  |  |  |  |  |  |  |  |  |  |  |  |
| Parent v. autistic adult | -.022 | .777 |  | -.193 | .009 |  | .075 | .355 |  | -.018 | .830 |  | -.155 | .054 |  | .073 | .390 |  | -.068 | .388 |
| Professional v. autistic adult | -.010 | .901 |  | -.287 | **<.001** |  | .117 | .181 |  | -.066 | .471 |  | -.237 | **.006** |  | -.063 | .490 |  | -.279 | **<.001** |
| Country |  |  |  |  |  |  |  |  |  |  |  |  |  |  |  |  |  |  |  |  |
| Aus. v. NZ | -.089 | .132 |  | -.029 | .604 |  | -.021 | .733 |  | -.080 | .214 |  | -.039 | .519 |  | .001 | .982 |  | -.083 | .161 |
| Gender |  |  |  |  |  |  |  |  |  |  |  |  |  |  |  |  |  |  |  |  |
| Female v. male | .038 | .577 |  | .020 | .759 |  | .040 | .579 |  | -.089 | .246 |  | .068 | .336 |  | -.145 | .055 |  | -.004 | .955 |
| Gender diverse v. male | .095 | .166 |  | .072 | .266 |  | -.001 | .992 |  | -.144 | .057 |  | .041 | .549 |  | -.150 | .044 |  | -.007 | .922 |
| Ethnicity |  |  |  |  |  |  |  |  |  |  |  |  |  |  |  |  |  |  |  |  |
| Māori v. NZ/Aus. European | -.133 | .019 |  | -.068 | .192 |  | -.066 | .259 |  | -.069 | .264 |  | -.075 | .182 |  | -.064 | .293 |  | -.067 | .238 |
| Asian v. NZ/Aus. European | -.130 | .023 |  | -.043 | .409 |  | -.034 | .563 |  | -.036 | .562 |  | -.006 | .914 |  | -.097 | .113 |  | -.006 | .918 |
| Pacific v. NZ/Aus. European | -.076 | .171 |  | -.085 | .108 |  | -.145 | .014 |  | -.073 | .235 |  | -.031 | .579 |  | -.055 | .370 |  | -.020 | .726 |
| Other v. NZ/Aus. European | .017 | .755 |  | -.042 | .422 |  | -.025 | .658 |  | .004 | .952 |  | .000 | 1.000 |  | .018 | .764 |  | .037 | .512 |
| Level of formal education |  |  |  |  |  |  |  |  |  |  |  |  |  |  |  |  |  |  |  |  |
| Undergraduate v. high school | -.031 | .687 |  | .168 | .026 |  | .137 | .098 |  | .141 | .103 |  | .128 | .114 |  | .008 | .928 |  | .045 | .573 |
| Postgraduate v. high school | .057 | .503 |  | .191 | .020 |  | .260 | **.004** |  | .181 | .054 |  | .256 | **.004** |  | .161 | .092 |  | .158 | .072 |
| Trade/Vocational v. high school | -.014 | .834 |  | -.016 | .795 |  | .052 | .458 |  | -.015 | .842 |  | .053 | .436 |  | .013 | .864 |  | .026 | .708 |
| Other v. high school | -.138 | .021 |  | .006 | .921 |  | .076 | .227 |  | .086 | .194 |  | .040 | .514 |  | -.022 | .732 |  | -.006 | .921 |
| Experience with young children |  |  |  |  |  |  |  |  |  |  |  |  |  |  |  |  |  |  |  |  |
| Some v. little | -.023 | .821 |  | -.062 | .517 |  | -.026 | .806 |  | .025 | .820 |  | -.049 | .623 |  | .145 | .180 |  | .019 | .847 |
| A lot v. little | -.009 | .932 |  | -.040 | .682 |  | .169 | .112 |  | .152 | .177 |  | .006 | .955 |  | .250 | .025 |  | .032 | .751 |
| **Perspectives on early support** |  |  |  |  |  |  |  |  |  |  |  |  |  |  |  |  |  |  |  |  |
| Appropriateness |  |  |  |  |  |  |  |  |  |  |  |  |  |  |  |  |  |  |  |  |
| Not appropriate v. appropriate | .065 | .248 |  | .092 | .085 |  | -.085 | .139 |  | -.053 | .384 |  | .080 | .159 |  | .033 | .590 |  | .054 | .339 |
| Depends v. appropriate | .345 | **<.001** |  | .360 | **<.001** |  | .224 | **<.001** |  | .183 | .008 |  | .336 | **<.001** |  | .250 | **<.001** |  | .303 | **<.001** |
| Model of support |  |  |  |  |  |  |  |  |  |  |  |  |  |  |  |  |  |  |  |  |
| Social v. medical | .189 | .063 |  | .361 | **<.001** |  | .216 | .041 |  | .298 | **.007** |  | .343 | **<.001** |  | .298 | **.007** |  | .348 | **<.001** |
| Biopsychosocial v. medical | .129 | .282 |  | .221 | .054 |  | .156 | .207 |  | .142 | .270 |  | .334 | **.005** |  | .293 | .022 |  | .229 | .053 |
| Strengths/difficulties v. medical | .106 | .383 |  | .249 | .029 |  | .107 | .387 |  | .133 | .299 |  | .303 | .011 |  | .276 | .031 |  | .221 | .063 |
| Other v. medical | .070 | .283 |  | .055 | .360 |  | .080 | .213 |  | -.056 | .404 |  | .109 | .093 |  | .030 | .665 |  | .092 | .160 |

Note: v. = versus; Aus. = Australia; NZ = New Zealand; Regression coefficients are based on Model 2, which includes all predictors. To control for Type 1 error across analyses, significance was set at p ≤ 0.007.
Communication: F(21, 266) = 4.340, p < 0.001; R^2^ (Model 1) = 0.115; R^2^ (Model 2) = 0.140; Cumulative R^2^ = 0.256
Autism characteristics: F(21, 234) = 8.517, p < 0.001; R^2^ (Model 1) = 0.226; R^2^ (Model 2) = 0.207; Cumulative R^2^ = 0.433
Academic skills: F(21, 254) = 3.722, p < 0.001; R^2^ (Model 1) = 0.148; R^2^ (Model 2) = 0.087; Cumulative R^2^ = 0.235
Motor skills: F(21, 246) = 2.527, p = 0.001; R^2^ (Model 1) = 0.071; R^2^ (Model 2) = 0.106; Cumulative R^2^ = 0.177
Play skills: F(21, 245) = 5.260, p < 0.001; R^2^ (Model 1) = 0.143; R^2^ (Model 2) = 0.168; Cumulative R^2^ = 0.311
Daily living skills: F(21, 250) = 2.662, p < 0.001; R2 (Model 1) = 0.078; R^2^ (Model 2) = 0.105; Cumulative R^2^ = 0.183
Participation: F(21, 249) = 5.045, p < 0.001; R^2^ (Model 1) = 0.138; R^2^ (Model 2) = 0.161; Cumulative R^2^ = 0.298

Table S8.

*Hierarchical linear regression predicting mean priority scores across goal domains for demographic characteristics unique to autistic adults*

|  | Communication | |  | Autism  characteristics | |  | Academic  skills | |  | Motor skills | |  | Play skills | |  | Daily living skills | |  | **Participation** | |
| --- | --- | --- | --- | --- | --- | --- | --- | --- | --- | --- | --- | --- | --- | --- | --- | --- | --- | --- | --- | --- |
| Predictors | β | *p* |  | β | *p* |  | β | *p* |  | β | *p* |  | β | *p* |  | β | *p* |  | β | *p* |
| Age |  |  |  |  |  |  |  |  |  |  |  |  |  |  |  |  |  |  |  |  |
| 26-30 v. 18-25 | -.004 | .980 |  | .117 | .503 |  | .037 | 0.821 |  | -.129 | .425 |  | .167 | .314 |  | .132 | .415 |  | .295 | .081 |
| 31-40 v. 18-25 | -.119 | .533 |  | .144 | .467 |  | .286 | 0.128 |  | .098 | .591 |  | .252 | .177 |  | .298 | .099 |  | .298 | .114 |
| >40 v. 18-25 | -.060 | .829 |  | .176 | .531 |  | .076 | 0.769 |  | -.053 | .836 |  | .215 | .415 |  | .160 | .526 |  | .281 | .294 |
| Age of diagnosis | .300 | .203 |  | .189 | .426 |  | .302 | 0.167 |  | .421 | .056 |  | .164 | .464 |  | .233 | .280 |  | .092 | .686 |
| Cooccurring diagnoses |  |  |  |  |  |  |  |  |  |  |  |  |  |  |  |  |  |  |  |  |
| Anxiety | -.099 | .557 |  | -.059 | .732 |  | .106 | 0.507 |  | -.098 | .536 |  | -.122 | .460 |  | .050 | .762 |  | -.034 | .836 |
| ADHD | .023 | .877 |  | .064 | .671 |  | -.043 | 0.751 |  | -.070 | .619 |  | .046 | .749 |  | -.066 | .622 |  | .014 | .923 |
| Depression | .046 | .770 |  | -.056 | .723 |  | .026 | 0.858 |  | .145 | .316 |  | -.015 | .922 |  | -.062 | .693 |  | -.049 | .748 |

Note: To control for Type 1 error across analyses, significance was set at p ≤ 0.007.
Communication: F(7,56) = 0.659; p = 0.659; R^2^ = 0.082
Autism characteristics: F(7,52) = 1.041; p = 0.415; R^2^ = 0.123
Academic skills: F(7,55) = 1.828, p = 0.100 ; R^2^ = 0.186
Motor skills: F(7,54) = 2.326, p = 0.038; R^2^ = 0.232
Play skills: F(7,55) = 1.359, p = 0.241; R^2^ = 0.147
Daily living skills: F(7,55) = 1.703, p = 0.127; R^2^ = 0.178

Participation: F(7,56) = 1.219, p = 0.308; R^2^ = 0.132

Table S9.

*Hierarchical linear regression predicting mean priority scores across goal domains for demographic characteristics unique to parents*

|  | Communication | |  | Autism  characteristics | |  | Academic  skills | |  | Motor skills | |  | Play skills | |  | Daily living skills | |  | Participation | |
| --- | --- | --- | --- | --- | --- | --- | --- | --- | --- | --- | --- | --- | --- | --- | --- | --- | --- | --- | --- | --- |
| Predictors | β | *p* |  | β | *p* |  | β | *p* |  | β | *p* |  | β | *p* |  | β | *p* |  | β | *p* |
| Relationship to child |  |  |  |  |  |  |  |  |  |  |  |  |  |  |  |  |  |  |  |  |
| Father v. mother | -.063 | .519 |  | -.028 | .770 |  | -.078 | 0.448 |  | .014 | .890 |  | -.047 | .636 |  | .054 | 0.584 |  | -.042 | .670 |
| Other v. mother | -.043 | .666 |  | -.029 | .765 |  | .077 | 0.452 |  | -.015 | .882 |  | .024 | .812 |  | -.003 | 0.973 |  | -.025 | .804 |
| Child gender |  |  |  |  |  |  |  |  |  |  |  |  |  |  |  |  |  |  |  |  |
| Male v. female | .055 | .558 |  | .126 | .178 |  | .059 | 0.552 |  | .093 | .350 |  | -.028 | .771 |  | .123 | 0.202 |  | .163 | .087 |
| Child age | .051 | .749 |  | -.122 | .405 |  | -.175 | 0.278 |  | -.158 | .328 |  | .038 | .806 |  | -.055 | 0.715 |  | -.155 | .306 |
| Child age of diagnosis | -.117 | .416 |  | .167 | .225 |  | .011 | 0.941 |  | .025 | .863 |  | -.173 | .228 |  | .117 | 0.41 |  | .062 | .663 |
| Child cooccurring conditions |  |  |  |  |  |  |  |  |  |  |  |  |  |  |  |  |  |  |  |  |
| Anxiety | -.182 | .142 |  | -.092 | .465 |  | -.095 | 0.497 |  | .089 | .523 |  | -.058 | .657 |  | .023 | 0.859 |  | -.070 | .585 |
| ADHD | .035 | .746 |  | -.093 | .396 |  | -.021 | 0.856 |  | .083 | .473 |  | -.109 | .341 |  | -.062 | 0.583 |  | -.082 | .464 |
| Depression | .063 | .583 |  | -.015 | .898 |  | .044 | 0.723 |  | -.025 | .846 |  | .082 | .506 |  | .008 | 0.951 |  | .113 | .349 |
| Global developmental delay | -.119 | .252 |  | -.219 | .035 |  | -.220 | 0.05 |  | -.246 | .030 |  | -.333 | **.002** |  | -.302 | **0.005** |  | -.196 | .065 |
| Intellectual and  developmental disability | .052 | .599 |  | -.025 | .800 |  | .040 | 0.702 |  | -.034 | .751 |  | -.099 | .336 |  | -.053 | 0.601 |  | .053 | .599 |
| Other cooccurring diagnoses | -.199 | .035 |  | .059 | .529 |  | -.107 | 0.281 |  | -.017 | .868 |  | .016 | .871 |  | .084 | 0.385 |  | .151 | .115 |
| Support >5 years |  |  |  |  |  |  |  |  |  |  |  |  |  |  |  |  |  |  |  |  |
| Any support | -.204 | .207 |  | -.058 | .711 |  | -.097 | 0.562 |  | -.251 | .134 |  | .056 | .729 |  | -.121 | 0.452 |  | .073 | .643 |
| Behavioural support | .038 | .713 |  | .084 | .381 |  | -.039 | 0.706 |  | .004 | .971 |  | .140 | .165 |  | .193 | 0.053 |  | .260 | .009 |
| Music therapy | .024 | .823 |  | -.080 | .454 |  | -.013 | 0.913 |  | -.009 | .935 |  | -.069 | .537 |  | .037 | 0.74 |  | .028 | .795 |
| Occupational therapy | .052 | .692 |  | .049 | .697 |  | .132 | 0.322 |  | .050 | .707 |  | .118 | .363 |  | .141 | 0.275 |  | .019 | .882 |
| Parent support | .172 | .107 |  | -.088 | .402 |  | .001 | 0.99 |  | -.066 | .553 |  | -.105 | .338 |  | -.190 | 0.082 |  | -.166 | .123 |
| Physiotherapy | .060 | .550 |  | -.010 | .918 |  | .060 | 0.562 |  | -.044 | .676 |  | .025 | .802 |  | .040 | 0.687 |  | -.038 | .701 |
| Psychologist support | .145 | .285 |  | -.064 | .635 |  | -.033 | 0.822 |  | .009 | .949 |  | .009 | .948 |  | .030 | 0.83 |  | -.179 | .192 |
| Social skills support | -.174 | .094 |  | -.180 | .077 |  | .015 | 0.892 |  | .018 | .865 |  | -.059 | .576 |  | -.113 | 0.279 |  | -.068 | .507 |
| Speech language therapy | -.010 | .948 |  | -.181 | .207 |  | -.230 | 0.135 |  | .029 | .854 |  | -.170 | .253 |  | -.019 | 0.899 |  | -.299 | .043 |
| Support ≤ 5 years |  |  |  |  |  |  |  |  |  |  |  |  |  |  |  |  |  |  |  |  |
| Any support | -.115 | .458 |  | .200 | .188 |  | .130 | 0.426 |  | .386 | .019 |  | .083 | .595 |  | .305 | 0.053 |  | .098 | .529 |
| Behavioural support | -.094 | .399 |  | -.037 | .734 |  | -.029 | 0.813 |  | -.025 | .836 |  | -.149 | .188 |  | -.056 | 0.615 |  | -.073 | .512 |
| Occupational therapy | .190 | .188 |  | .050 | .720 |  | -.102 | 0.489 |  | -.106 | .493 |  | -.155 | .281 |  | -.063 | 0.66 |  | .007 | .962 |
| Psychologist support | .046 | .673 |  | .094 | .369 |  | .007 | 0.947 |  | -.061 | .592 |  | .123 | .259 |  | .076 | 0.482 |  | .094 | .382 |
| Social skills support | .077 | .468 |  | -.002 | .989 |  | -.028 | 0.803 |  | -.010 | .930 |  | -.056 | .606 |  | .011 | 0.919 |  | -.070 | .512 |
| Speech language therapy | -.186 | .180 |  | -.267 | .048 |  | -.104 | 0.471 |  | -.225 | .137 |  | -.148 | .289 |  | -.198 | 0.154 |  | -.130 | .355 |

Note: To control for Type 1 error across analyses, significance was set at p ≤ 0.007.
Communication: F(26,112) = 0.815; R^2^ = 0.146
Autism characteristics: F(26,102) = 1.427, p = 0.108; R^2^ = 0.267
Academic skills: F(26,103) = 0.697, p = 0.854; R^2^ = 0.150
Motor skills: F(26,98) = 0.871, p = 0.645; R^2^ = 0.188
Play skills: F(26, 100) = 1.079, p = 0.379; R^2^ = 0.219
Daily living skills: F(26,102) = 1.096, p = 0.361; R^2^ = 0.218
Participation: F(26,100) =1.265, p = 0.204; R^2^ = 0.247

Table S10.

*Hierarchical linear regression predicting mean priority scores across goal domains for demographic characteristics unique to professionals*

|  | Communication | |  | Autism  characteristics | |  | Academic  skills | |  | Motor skills | |  | Play skills | |  | Daily living skills | |  | Participation | |
| --- | --- | --- | --- | --- | --- | --- | --- | --- | --- | --- | --- | --- | --- | --- | --- | --- | --- | --- | --- | --- |
| Predictors | β | *p* |  | β | *p* |  | β | *p* |  | β | *p* |  | β | *p* |  | β | *p* |  | β | *p* |
| Role |  |  |  |  |  |  |  |  |  |  |  |  |  |  |  |  |  |  |  |  |
| Speech language therapist | .083 | .514 |  | .071 | 0.59 |  | -.015 | .906 |  | -.014 | .913 |  | .090 | .504 |  | .156 | .234 |  | .132 | .318 |
| Psychologist | .044 | .726 |  | -.101 | 0.434 |  | .016 | .901 |  | .014 | .913 |  | -.077 | .562 |  | -.049 | .703 |  | -.124 | .341 |
| Years in role | -.062 | .695 |  | .148 | 0.346 |  | .073 | .642 |  | .243 | .121 |  | .149 | .353 |  | .117 | .455 |  | .010 | .947 |
| Years of experience autism | .133 | .403 |  | -.125 | 0.417 |  | .178 | .256 |  | .095 | .543 |  | -.069 | .659 |  | -.011 | .943 |  | .000 | .999 |

Note: To control for Type 1 error across analyses, significance was set at p ≤ 0.007.
Communication: F(4,64) = 0.345, p = 0.847; R^2^ = 0.021
Autism characteristics: F(4,61) = 0.513, p = 0.726; R^2^  = 0.033
Academic skills: F(4,63) = 0.883, p = 0.479; R^2^ = 0.053
Motor skills: F(4,61) = 1.614, p = 0.182; R^2^ = 0.096
Play skills: F(4,58) = 0.423, p = 0.791; R^2^ = 0.028
Daily living skills: F(4,61) = 0.579, p = 0.679; R^2^ = 0.037
Participation: F(4,60) = 0.594; p = 0.669; R^2^ = 0.038
